# Molecular Characterization of Microorganisms, Pectinolytic Potential and Volatile Compound Formation During Spontaneous Fermentation of Criollo Cacao (*Theobroma cacao* L.)

**DOI:** 10.3390/foods15193462

**Published:** 2026-09-28

**Authors:** Lisset Ll. Alvites-Chuquizuta, Angel David Hernández-Amasifuen, Harvey Pérez-Ramos, Erick A. Auquiñivin-Silva, Luz Quispe-Sanchez, Marleni Medina-Mendoza, Roberth Esteve Iliquin-Fernandez, Laydy Mitsu Mena-Chacon, Carlos Culqui-Arce, Robert J. Cruzalegui, Diner Mori-Mestanza

**Affiliations:** 1 Instituto de Investigación, Innovación y Desarrollo para el Sector Agrario y Agroindustrial (IIDAA), Facultad de Ingeniería y Ciencias Agrarias, Universidad Nacional Toribio Rodríguez de Mendoza de Amazonas, Chachapoyas 01000, Peru; 7486858292@untrm.edu.pe (L.L.A.-C.); angel.hernandez@untrm.edu.pe (A.D.H.-A.); harvey.perez@untrm.edu.pe (H.P.-R.); carlos.culqui@untrm.edu.pe (C.C.-A.); robert.cruzalegui@untrm.edu.pe (R.J.C.); 2 Programa Doctoral en Ciencias para el Desarrollo Sustentable, Escuela de Posgrado, Facultad de Ingeniería Zootecnista, Biotecnología, Agronegocios y Ciencia de Datos, Universidad Nacional Toribio Rodríguez de Mendoza de Amazonas, Chachapoyas 01001, Peru; luz.quispe.epg@untrm.edu.pe (L.Q.-S.); marleni.medina@untrm.edu.pe (M.M.-M.); roberth.iliquin@untrm.edu.pe (R.E.I.-F.); laydy.mena@untrm.edu.pe (L.M.M.-C.)

**Keywords:** aromatic profile, spontaneous fermentation, culturable microorganisms, pectinolytic screening, volatile compounds, HS-SPME/GC-MS

## Abstract

Spontaneous fermentation of Criollo cacao involves temporal changes in culturable microorganisms and in the volatile compound profile, with potential implications for the development of aroma-related characteristics. This study aimed to characterize culturable microorganisms, assess their apparent pectinolytic potential, and evaluate the temporal dynamics of volatile compounds during spontaneous fermentation of Criollo cacao from the Lagunas rural community, Amazonas, Peru. Microorganisms were isolated at different fermentation stages, and pectinolytic potential was evaluated by screening for pectin-degradation halos on a mineral medium supplemented with pectin. Selected isolates were molecularly identified by sequencing the ITS region for yeasts and the 16S rRNA gene for bacteria. Volatile compounds were analyzed by headspace solid-phase microextraction coupled with gas chromatography–mass spectrometry (HS-SPME/GC-MS). All isolates evaluated in the pectin assay produced visible degradation halos, although halo diameter and area varied among isolates, indicating differences in apparent pectinolytic potential. Molecular characterization showed frequent recovery of *Pichia kudriavzevii*, *Saccharomyces cerevisiae*, and *Hanseniaspora opuntiae* among yeasts, whereas *Acetobacter* species were frequently recovered among bacteria. Volatile profiling revealed temporal changes in the relative abundance of alcohols, esters, aldehydes, ketones, terpenes, acids, and other compound families. Exploratory Spearman correlation analysis identified several strong temporal associations between microbial recovery frequency and VOC families, particularly involving *P. kudriavzevii*, *Acetobacter pasteurianus*, *A. tropicalis*, and *Bacillus subtilis*. However, these associations did not remain significant after correction for multiple comparisons. Overall, the integrated culture-dependent, molecular, pectinolytic screening, and volatile profiling approach provides preliminary evidence of coordinated temporal patterns between culturable microorganisms and volatile compounds during spontaneous Criollo cacao fermentation and identifies candidate isolates for further functional evaluation.

## 1. Introduction

Cacao (*Theobroma cacao* L.) is an economically important crop and the primary raw material for chocolate production. In northeastern Peru, Criollo, Forastero, Trinitario, and CCN-51 are cultivated, with Criollo valued for its bioactive composition and sensory attributes [1,2]. In the Amazonas Region, particularly in the provinces of Utcubamba and Bagua, this variety is an important crop for local production [3].

Spontaneous cocoa fermentation involves yeasts, lactic acid bacteria (LAB), and acetic acid bacteria (AAB). However, their relative abundance varies with cocoa genotype, fermentation conditions, geographical origin, and the analytical method used for microbial characterization [4,5,6]. Their metabolic activities transform sugars and other pulp components and contribute to the formation of volatile compounds and aroma precursors [7,8]. Because spontaneous fermentation is inherently variable, starter cultures and characterization of native microbiota are important strategies for improving fermentation consistency and cocoa quality [9,10].

Recent studies have shown that DNA-based molecular techniques, including high-throughput sequencing, allow a more comprehensive characterization of the microbial communities present during cacao fermentation and provide greater insight into their dynamics and potential metabolic functions [11,12]. Previous studies have also reported associations between microbial community composition and volatile compound production during cocoa fermentation [8,13].

However, information remains limited on the culturable microbiota and volatile profile of Criollo cacao fermented in the rural community of Lagunas, Cajaruro District, Utcubamba Province, Amazonas Region, Peru, an area recognized for the production of this variety. Therefore, this study aimed to characterize culturable microorganisms, evaluate their pectinolytic potential using an agar-based screening assay, and determine the temporal dynamics of volatile compounds during the spontaneous fermentation of Criollo cacao. The study provides preliminary evidence of temporal associations between the recovery of culturable microorganisms and changes in volatile profiles throughout fermentation.

## 2. Materials and Methods

### 2.1. Criollo Cacao Collection

Criollo cacao was obtained from the rural community of Lagunas, Cajaruro District, Utcubamba Province, Amazonas Region, Peru (5°45′40.9″ S, 78°15′17.1″ W; 849 m a.s.l.). A total of 1800 kg of freshly harvested Criollo cocoa beans was collected and distributed equally among three wooden fermentation boxes, with 600 kg allocated to each box. Fermentation was conducted simultaneously in three wooden boxes at the APROCAM Multiple Services Cooperative, located at km 4 of the Bagua–Copallín Highway, Bagua Province (5°39′24.23″ S, 78°29′50.17″ W; 478.63 m a.s.l.). The three boxes represented parallel fermentation units from the same lot. Fermentation was monitored for 144 h, and samples were collected at 0, 24, 48, 72, 96, 120, and 144 h. [14]. The sampling scheme is summarized in Figure 1.

Because the three boxes were filled from the same cocoa lot and fermented simultaneously under the same processing conditions, they were considered parallel fermentation units rather than independent biological replicates. Accordingly, temporal patterns observed in this study should be interpreted as descriptive of this fermentation trial and not as independently replicated fermentation events.

### 2.2. Isolation of Microorganisms from Criollo Cacao Fermentation

Potato dextrose agar (PDA) medium was prepared according to the methodology described by Laboratorios Microkit and Oblitas et al. [15,16]. The medium was poured into Petri dishes under a laminar flow cabinet and stored in expanded polystyrene containers with ice packs for transportation to the field.

Culturable microorganisms were recovered from cocoa samples collected throughout the fermentation process, from 0 to 144 h. Cocoa beans were rubbed with a bacteriological inoculating loop, and the collected material was streaked onto PDA plates using the zigzag technique described by Soto et al. [17]. The inoculated plates were kept refrigerated until transportation to the laboratory for subsequent analysis.

PDA plates inoculated with samples from each fermentation stage were incubated at 25–28 °C for 48–72 h. Incubation was performed using an INCUCELL 55–ECO incubator (BMT Medical Technology, Brno, Czech Republic) at the Universidad Nacional Toribio Rodríguez de Mendoza de Amazonas (UNTRM), Chachapoyas, Peru. These conditions were based on Figueroa et al. [18].

### 2.3. Preparation of Culture Media and Microbial Subculturing

Microbial isolation was initially performed on PDA plates to recover culturable microorganisms from fermenting cocoa bean samples. Morphologically distinct colonies were subsequently subcultured on YPD and MRS media for purification and characterization. YPD was used primarily for yeast recovery, whereas MRS was used primarily for bacterial recovery. Neither medium was considered strictly selective under the experimental conditions, and antimicrobial supplements were used only to limit the growth of non-target microorganisms. Accordingly, isolates were classified based on growth characteristics and subsequent molecular identification, regardless of the medium on which they grew. Bacterial growth on YPD and yeast growth on MRS were therefore regarded as cross-growth rather than evidence of medium specificity.

De Man, Rogosa, and Sharpe (MRS) medium was prepared according to previously described procedures [19,20], and supplemented with 1200 µL/L fluconazole before sterilization. Yeast peptone dextrose (YPD) medium was prepared according to the manufacturer’s formulation (Condalab, Madrid, Spain) [21]. YPD is routinely used for the cultivation and maintenance of yeasts [22] and was supplemented with 500 mg/L azithromycin under the same sterilization conditions described above.

The media were dispensed into tubes and allowed to solidify in an inclined position before inoculation. Morphologically distinct colonies recovered from PDA plates were transferred to three YPD and three MRS tubes per plate for purification and preliminary characterization. Cultures were incubated at 28 °C for 72 h [23]. Based on colony morphology and growth characteristics, 56 representative cultures were selected for molecular identification and stored at 4 °C until analysis.

### 2.4. Preparation of Mineral Medium and Assessment of Pectinolytic Potential

The pectinolytic potential of the 56 selected cultures (28 recovered from YPD and 28 from MRS) was evaluated using a qualitative halo-formation assay on pectin-containing mineral medium. The medium contained pectin (5 g/L), glucose (5 g/L), KH_2_PO_4_ (6 g/L), yeast extract (1 g/L), ammonium nitrate (2 g/L), and agar (15 g/L). A micronutrient solution was added at 0.1 mL/L and contained FeSO_4_ (0.001 g/L), MgSO_4_ (0.2 g/L), CaCl_2_ (0.001 g/L), H_3_BO_3_ (0.002 g/L), MnSO_4_ (0.002 g/L), ZnSO_4_·7H_2_O (0.014 g/L), CuSO_4_·5H_2_O (0.01 g/L), and MoO_3_ (0.002 g/L). The final pH was adjusted to 7.2. The formulation was based on conditions previously used to evaluate pectinolytic microorganisms associated with coffee [24].

For the assay, biomass from each selected culture was inoculated as a single central spot on the mineral medium. No defined biomass concentration or inoculum mass was used. The procedure followed a point-inoculation approach similar to that described by Xu et al. [25], with adaptations for the present assay. Three replicate plates were prepared per culture and incubated at 28 °C for 4 days.

After incubation, a visualization solution containing 1.4 M NaCl, 1% (*w*/*v*) cetyltrimethylammonium bromide (CTAB), 50 mM Tris-HCl, and 10 mM EDTA was applied to the plate surface. The solution remained in contact with the agar for 3 h to visualize pectin-degradation zones, following an approach similar to that reported by Fernandez–Güimac et al. [26]. A visible clearing zone surrounding the colony was considered indicative of pectinolytic potential.

The plates were photographed using a digital camera, and the images were analyzed with ImageJ v1.54p (Fiji distribution, National Institutes of Health, Bethesda, MD, USA). For each replicate, colony diameter and area and halo diameter and area were measured under standardized conditions. The halo-to-colony ratio (HCR) was calculated as

$$HCR=\frac{halo\;diameter}{colony\;diameter}$$

Isolates were compared descriptively based on halo formation, halo diameter and area, colony diameter and area, and HCR. Because the assay was based on halo formation on solid medium, it did not quantify extracellular enzyme concentration or catalytic activity. Therefore, HCR was used as a comparative indicator of apparent pectinolytic potential rather than as a direct measure of enzymatic activity or pectin degradation during cacao fermentation.

### 2.5. Preculture, Isolation, and Concentration of Microbial Biomass for DNA Extraction

The 56 selected microbial cultures were subjected to molecular identification. Of the 28 cultures recovered from YPD, 24 with yeast-like morphology were analyzed by ITS amplification and four with bacterial-like morphology by 16S rRNA gene amplification. The 28 cultures recovered from MRS were also analyzed by 16S rRNA gene amplification. In total, 24 isolates were analyzed using ITS and 32 using 16S rRNA gene amplification.

For DNA extraction, yeast isolates recovered from YPD were first streaked onto PDA plates and incubated at 25 °C for 5 days, under the conditions described by Maghradze et al. [27]. Bacterial isolates selected for 16S rRNA gene amplification were transferred to a liquid nutrient medium containing tryptone (4 g/L), yeast extract (2 g/L), and NaCl (2 g/L) and incubated at 20 °C and 180 rpm for 2 days in an orbital shaker-incubator (Biosan, ES-20/80, Riga, Latvia), following the conditions described by Zarei et al. [28].

Approximately 0.1 g of microbial biomass was collected from each selected culture for DNA extraction, following a procedure adapted from Hamilton et al. [29]. For bacterial isolates, cell biomass was first concentrated by centrifugation of the liquid cultures at 13,000 rpm for 3 min, and the resulting pellets were transferred to individual microcentrifuge tubes, following Hoorzook & Barnard [30].

### 2.6. DNA Isolation of Microorganisms

Genomic DNA was extracted from the selected yeast and bacterial isolates using a chemical lysis procedure based on 2× cetyltrimethylammonium bromide (CTAB), Tris-HCl, β-mercaptoethanol, chloroform, and isopropanol, following protocols similar to those described by Tiwari and Muthanna [31]. The extraction procedure was adapted to microbial biomass using the same chemical reagents and included cell lysis by incubation at 65 °C in a shaking thermoblock (ThermoMixer C, Eppendorf, Hamburg, Germany), followed by centrifugation and recovery of the aqueous phase. DNA was subsequently precipitated with isopropanol and stored at −20 °C until further processing.

The precipitated DNA was further purified by centrifugation and washing with 70% ethanol, following a procedure similar to that described by Hoorzook and Barnard [30]. The resulting DNA pellets were air-dried, rehydrated in molecular-grade water, and maintained under refrigeration for 24 h before subsequent analysis, following the procedure reported by Xie et al. [32].

DNA concentration and integrity were evaluated by electrophoresis on 1% agarose gels prepared in TAE buffer, following Parks and Torres [33]. Electrophoresis was performed at 100 V for 25 min, and DNA fragments were visualized under UV light using a chemiluminescence imaging documentation system. DNA preparations showing suitable integrity and concentration were used as templates for subsequent PCR amplification.

### 2.7. PCR Amplification and Molecular Identification

The internal transcribed spacer (ITS) region of the yeast isolates and the 16S rRNA gene of the bacterial isolates were amplified by polymerase chain reaction (PCR). The ITS region was amplified using the universal primers ITS1 (5′-TCCGTAGGTGAACCTGCGG-3′) and ITS4 (5′-TCCTCCGCTTATTGATATGC-3′), as described by White et al. [34]. For bacterial isolates, a fragment of the 16S rRNA gene was amplified using the universal primers 27F (5′-AGAGTTTGATCCTGGCTCAG-3′) and 1492R (5′-TACGGYTACCTTGTTACGACTT-3′), following Weisburg et al. [35]. Each PCR was performed in a final volume of 30 μL containing 50 ng of template DNA, 0.2 mM dNTPs, 1 μM of each primer, 1× PCR buffer, 1 U of Taq DNA polymerase, and molecular-grade water to the final volume.

PCR amplification was performed using a T100 Thermal Cycler (Bio-Rad Laboratories, Hercules, CA, USA). For amplification of the bacterial 16S rRNA gene, the thermal cycling program consisted of an initial denaturation at 95 °C for 5 min, followed by 35 cycles of denaturation at 95 °C for 30 s, annealing at 57 °C for 90 s, and extension at 72 °C for 2 min, with a final extension at 72 °C for 10 min. For amplification of the yeast ITS region, the program consisted of an initial denaturation at 95 °C for 5 min, followed by 35 cycles of denaturation at 95 °C for 40 s, annealing at 58 °C for 45 s, and extension at 72 °C for 2 min, followed by a final extension at 72 °C for 10 min.

PCR products were evaluated by electrophoresis on 1% agarose gels prepared in 1× TAE buffer and visualized using a ChemiDoc Imaging System (Bio-Rad Laboratories, Hercules, CA, USA). Amplicons displaying the expected amplification pattern were excised and purified using the QIAEX II Gel Extraction Kit (QIAGEN, Hilden, Germany), according to the manufacturer’s instructions. Purified amplicons were subsequently subjected to bidirectional Sanger sequencing by Macrogen Inc. (Seoul, Republic of Korea).

Forward and reverse chromatograms were inspected to assess sequence quality, and low-quality regions were trimmed before generating consensus sequences using BioEdit. After sequence processing, consensus sequences of approximately 970 bp for the bacterial 16S rRNA gene and 650 bp for the yeast ITS region were obtained. The consensus sequences were compared with nucleotide sequences deposited in the NCBI GenBank database using BLASTn (BLAST+ v2.15.0). Species-level assignments were considered putative. They were based on the closest GenBank matches showing ≥98% nucleotide identity, together with the highest sequence similarity and query coverage.

### 2.8. Analysis of Volatile Compounds by HS-SPME/GC–MS

Volatile organic compounds (VOCs) were extracted by headspace solid-phase microextraction (HS-SPME) using a 50/30 μm divinylbenzene/carboxen/polydimethylsiloxane (DVB/CAR/PDMS) fiber (Supelco). Three sample matrices were analyzed independently: (i) 2 g of cocoa beans collected at each fermentation time, (ii) 2 g of cocoa testa collected throughout the fermentation process, and (iii) 5 g of agar containing microbial growth associated with cocoa fermentation, as described in Section 2.3.

For bean and testa samples, the material was cryogenically ground in a porcelain mortar using liquid nitrogen. The homogenized samples were then transferred separately to 20 mL headspace vials and sealed with aluminum caps fitted with polytetrafluoroethylene (PTFE)/silicone septa. Samples were collected at 0 h immediately after pod opening and at 0, 24, 48, 72, 96, 120, and 144 h of fermentation. The initial sample obtained immediately after pod opening was designated as 0 h/pod.

For microbial samples, one culture from each fermentation time was selected according to its performance in the pectinolytic screening assay, considering the halo-to-colony ratio (HCR). The selected microbial cultures were analyzed as representatives of isolates showing the relevant pectinolytic screening response.

Before VOC extraction, the headspace of each vial was equilibrated at 60 °C for 15 min. followed by exposure of the fiber to the headspace at the same temperature for 30 min using a digital heating block (Model No. 949VWHB2EUA, VWR, Radnor, PA, USA). After extraction, the fiber was inserted into the injection port of a gas chromatography–mass spectrometry system (7890B GC System and 5977B MSD, Agilent Technologies, Santa Clara, CA, USA).

Chromatographic separation was performed using a DB-5MS UI capillary column (60 m × 0.25 µm × 1.0 μm, Agilent). The oven temperature was initially maintained at 50 °C for 5 min and then increased at a rate of 4 °C/min until reaching 250 °C. Helium was used as the carrier gas at a constant flow rate of 1.1 mL/min. The injector, operated in split mode at a 1:1 ratio, transfer line, ion source, and quadrupole temperatures were maintained at 250, 250, 230, and 150 °C, respectively. Data were acquired in scan mode using electron impact ionization at 70 eV over a mass range of 35–400 amu [36]. Additionally, a homologous mixture of n-alkanes (C8–C20) was analyzed under the same chromatographic conditions as the samples to calculate the linear retention indices (LRIs) of the detected volatile compounds [37].

Chromatographic peaks were deconvoluted using Agilent MassHunter Unknowns Analysis (version 10.1 build 10.1.733.0, Agilent Technologies). VOCs were tentatively identified by comparison of their mass spectra with entries in the NIST 17 mass spectral library (Gaithersburg, MD, USA) and by comparison of their calculated LRIs with literature values. Identifications supported by both a mass spectral match factor ≥70% and agreement between the calculated and literature LRI values, with a deviation of no more than ±40 LRI units, were classified as MS + LRI.

Because authentic standards and compound-specific calibration curves were not used, the detected VOCs were reported as tentative identifications and their abundances were expressed relatively rather than as absolute concentrations. Accordingly, differences among samples were interpreted as changes in relative abundance within the analytical profiles and not as quantitative changes in compound concentration.

### 2.9. Statistical Analysis

Data analysis was performed using R software (version 4.5.3). Pectinolytic screening data were summarized using descriptive statistics and visualized with box-and-whisker plots. The halo-to-colony ratio was used as a comparative indicator of apparent pectinolytic potential. Microbial isolation data were summarized as recovery frequencies at each fermentation time and visualized using heat maps.

The three fermentation boxes originated from the same cocoa lot and were operated simultaneously as parallel units within a single trial. Therefore, they were not considered independent biological replicates, and temporal patterns in microbial recovery and VOC profiles were analyzed descriptively rather than inferentially.

Associations between microbial recovery frequency and the relative abundance of VOC families were explored using Spearman’s rank correlation coefficient (ρ) across seven fermentation stages (0–144 h). A total of 132 microorganism–VOC family comparisons were performed. *p*-values were adjusted using the Benjamini–Hochberg procedure to control the false discovery rate (FDR). Adjusted *p*-values < 0.05 were considered statistically significant, whereas nominal *p*-values < 0.05 were reported only as exploratory associations. Because microbial data were culture-dependent, correlations were interpreted as temporal associations rather than evidence of microbial abundance, community succession, or causality.

Multivariate patterns in VOC families were explored using principal component analysis (PCA) and cluster analysis to characterize variation and sample grouping. No inferential significance was assigned to differences among fermentation times because independent biological replication was not available.

## 3. Results and Discussion

### 3.1. Morphological Analysis and Pectin Degradation Halos of Microorganisms Isolated from Fermented Cacao Beans

The formation of clear halos around the colonies provided qualitative evidence of apparent pectinolytic potential among the culturable isolates recovered during spontaneous fermentation [38]. Pectin degradation in the assay reflects the capacity of isolates to produce extracellular enzymes that hydrolyze pectin under the tested in vitro conditions. However, halo formation should be interpreted as a preliminary screening indicator rather than as a direct measure of enzymatic activity or pectin degradation during cacao fermentation [5,39].

After incubation, all isolates developed visible degradation halos, with halo areas ranging from 41.01 to 63.64 cm^2^. These differences showed variation in halo formation among isolates under the in vitro assay conditions. Because halo size can be influenced by colony growth and enzyme diffusion, the halo-to-colony diameter ratio was also used as a comparative screening parameter. According to Ortiz Galeano et al. [40], larger degradation zones are commonly associated with greater apparent pectinolytic potential in plate-based screening assays. However, halo size may also be affected by colony growth, enzyme diffusion, and the physicochemical properties of the assay medium. Therefore, differences in halo size should be interpreted cautiously when comparing isolates. Previous studies have shown that pectinolytic activity can be influenced by environmental and nutritional conditions, including pH, temperature, and carbohydrate availability [12,41]. However, these factors were not directly evaluated in the present assay.

Most isolates exhibited intermediate halo-forming capacity, whereas only a small number produced halos larger than 60 cm^2^, indicating relatively larger degradation zones under the in vitro screening conditions. This pectinolytic capacity may contribute to mucilage degradation and changes in the physical environment of the fermenting mass. Such changes have been associated in previous studies with the establishment and activity of bacterial groups involved in cacao fermentation and the formation of aroma-related metabolites [42].

Figure 2 shows yeast colonies isolated at the beginning (0 h) and end (144 h) of the spontaneous cacao fermentation process, before (Figure 2A, 0 h; Figure 2A, 144 h) and after (Figure 2B, 0 h; Figure 2B, 144 h) the addition of 1% CTAB. At 0 h, the higher colony density may be related to the availability of fermentable substrates in fresh cocoa mucilage. Previous studies have reported favorable conditions for yeast growth during the early stages of cacao fermentation [5,39]. Later stages are generally associated with substrate depletion, ethanol accumulation, organic acid production, and temperature changes. However, temperature and pH were not monitored in the present study. These variables therefore cannot be used to directly explain the recovery patterns observed here.

Such changes have been associated in previous studies with reduced yeast growth and increased recovery of acid-tolerant bacterial groups during cacao fermentation [42,43]. However, lactic acid bacteria (LAB) were not identified among the isolates analyzed in the present study. Given the culture-dependent design and the isolation conditions used, this finding should not be interpreted as evidence that LAB were absent from the fermentation ecosystem.

Following the addition of CTAB, clear degradation halos were observed around the colonies, indicating detectable pectin-degrading potential among some yeast isolates under the in vitro assay conditions. This finding identifies isolates that may warrant further investigation for their pectinolytic potential and possible technological relevance in cacao fermentation [12,41].

Figure 3 shows the bacterial colonies isolated at the beginning (0 h) and end (144 h) of the spontaneous cacao fermentation process, before (3A, 0 h; 3A, 144 h) and after (3B, 0 h; 3B, 144 h) the addition of 1% CTAB. In contrast to yeasts (Figure 2), bacteria exhibited moderate growth during the initial stages because yeasts were frequently recovered during the early stages of fermentation, which consume mucilage sugars and produce ethanol, thereby progressively creating favorable conditions for the establishment of bacterial populations [12,44]. As fermentation progressed, changes in the frequency of recovery of culturable bacterial isolates were observed. Acetic acid bacteria were recovered at several fermentation stages, whereas other bacterial taxa were detected less frequently or mainly during later stages. These temporal patterns are consistent with the ecological and metabolic roles reported for bacterial groups involved in cacao fermentation [45,46]. However, because the present study relied on culture-dependent isolation, these observations should not be interpreted as evidence of changes in the total bacterial community or as direct evidence of temporal changes in the recovery frequency of culturable microorganisms.

Following the addition of CTAB, degradation halos were observed around the bacterial colonies, indicating pectin-degrading potential under the in vitro screening conditions. The presence of these halos demonstrates the ability of the bacteria to produce enzymes that hydrolyze the pectin present in the mucilage, thereby contributing to its degradation even when the yeast population declines [12]. Pectin hydrolysis may facilitate the release of soluble substrates that can subsequently participate in biochemical transformations associated with cacao fermentation [44,46]. However, the present study did not directly measure nutrient release or sensory quality.

The greater definition of the halos at 144 h indicates that some bacterial isolates recovered at this stage exhibited comparatively greater halo-forming capacity under the in vitro screening conditions. Previous studies have reported that the later stages of cacao fermentation are commonly associated with increased temperature and acidification resulting from microbial metabolism [45]. Such conditions may favor microorganisms with greater tolerance to thermal and acidic stress [41]. However, these physicochemical parameters were not measured in the present study and therefore cannot be used as direct evidence to explain the recovery patterns observed here.

The variability observed in colony morphology was accompanied by differences in halo formation among the bacterial isolates. These observations indicate that pectin-degrading potential was detected among multiple culturable bacterial isolates recovered throughout fermentation [12,41]. Figures S1 and S2 provide additional morphological and halo-formation data for the individual isolates.

Figure 4 presents the boxplot of the halo-to-colony diameter ratio for yeast isolates during the spontaneous fermentation of Criollo cacao. This analysis is complemented by the line plots included in Figures S3 and S4, which support the temporal trends observed throughout the fermentation process.

Differences among fermentation times indicate that yeast pectinolytic potential responded to physicochemical changes in the fermenting mass. During the initial stages, the high availability of sugars in the mucilage, along with environmental conditions widely documented in previous studies, such as temperatures of approximately 30–35 °C and pH values ranging from 3.5 to 4.5, favors yeast growth and the production of pectinolytic enzymes associated with pectin degradation and mucilage drainage [7,47]. Likewise, the intense metabolic potential of these microorganisms promotes the production of ethanol and other metabolites that initiate the temporal changes in the recovery frequency of culturable microorganisms characteristic of cacao fermentation [48,49].

As fermentation progressed, pectinolytic potential showed greater variability among the isolates, which was associated with physiological differences within the microbiota in response to the changing process conditions. Previous studies have shown that depletion of fermentable sugars, ethanol accumulation, and subsequent acetic acid production can alter the physicochemical environment of cacao fermentation [44,49]. These changes have been associated with increased acid and thermal stress, which may influence the recovery of culturable yeasts during fermentation [7,47].

During the final stages of fermentation, the lower halo-to-colony ratios observed for some yeast isolates may be associated with changes in substrate availability and other environmental stresses reported for cacao fermentation [44]. Acetic acid production by acetic acid bacteria has also been associated with increasing acid and thermal stress during the later stages of fermentation [7,48]. These results explain the reduced pectinolytic halo diameters observed in specific isolates. They further indicate that yeast enzymatic screening profiles correspond to the temporal variation in cultivable microbial recovery throughout spontaneous cacao fermentation [49].

Figure 5 presents the boxplot of the halo-to-colony diameter ratio for bacterial isolates cultured on MRS medium during the spontaneous fermentation of Criollo cacao. This analysis is complemented by the line plots shown in Figures S3 and S4, which support the trends observed in apparent pectinolytic potential throughout the fermentation process.

During the first 24 h, the areas and diameters of both the degradation halos and colonies were low, indicating limited bacterial pectinolytic potential. This behavior coincides with the early stage of cacao fermentation, during which yeasts are commonly recovered and fermentable sugars are readily available. Such conditions have been reported to favor yeast growth during the early stages of cacao fermentation [5,50]. The progressive modification of the fermentation environment through ethanol production and mucilage degradation subsequently favors the development of bacteria associated with cacao fermentation [51].

From 48 h onward, an increase in both halo and colony dimensions was observed, reaching maximum values between 72 and 96 h. Under these in vitro screening conditions, bacterial isolates recovered during these intermediate stages exhibited a higher halo-forming capacity. Notably, although lactic acid bacteria (LAB) are frequently reported during the mid-stage of cacao fermentation, no LAB were isolated in the present study despite the use of MRS medium. This outcome aligns with known culture-dependent constraints, whereby antimicrobial supplementation or rapid overgrowth by acetic acid bacteria (AAB) can suppress LAB recovery [52]. Instead, the isolates recovered at this stage were predominantly identified as Acetobacter and Bacillus. Members of the genus Acetobacter are well established as key drivers of ethanol oxidation to acetic acid during cacao fermentation [5,51]. As widely documented in the cacao fermentation literature, this accumulation of acetic acid induces mass acidification and a rise in temperature, which in turn facilitates the diffusion of organic acids into the cotyledons and fosters the development of essential flavor precursors [50,53].

The larger pectinolytic halos observed between 120 and 144 h, consistent with the trends in Figures S3 and S4, demonstrate that specific bacterial isolates maintained halo-forming capacity under these in vitro screening conditions. These findings suggest a potential contribution of bacterial strains to mucilage degradation during the final stages of fermentation. Such degradation could drive environmental changes in the fermenting mass that indirectly influence metabolite production and subsequent flavor development [50,52].

### 3.2. Molecular Identification of Yeasts and Bacteria During the Spontaneous Fermentation of Criollo Cacao

Sequencing of the ITS region enabled the identification of three yeast species associated with the spontaneous fermentation of Criollo cacao: *Pichia kudriavzevii*, *Saccharomyces cerevisiae*, and *Pichia manshurica* (Table 1). *P*. *kudriavzevii* was isolated most frequently during the early stages of fermentation (0–72 h), whereas *S. cerevisiae* was recovered mainly between 72 and 120 h, and *P*. *manshurica* was detected at 120 h. These observations indicate temporal changes in the frequency of recovery of culturable yeasts during fermentation rather than changes in their actual population abundance. In addition, an isolate recovered on MRS medium at 96 h was identified as *S. cerevisiae*, indicating that this species was culturable at different fermentation stages [54].

The higher frequency of recovery of *P*. *kudriavzevii* during the early stages is consistent with previous studies in which this species was frequently isolated from cacao fermentation during the initial stages [55,56]. Subsequently, the presence of *S*. *cerevisiae* between 72 and 120 h agrees with previous studies describing its involvement in the active fermentation phase and its contribution to ethanol production, thereby promoting microbiological changes throughout the process [55]. Finally, the detection of *P*. *manshurica* during the final stages suggests greater tolerance to fermentation conditions, a behavior similar to that reported for yeasts of the genus *Pichia* associated with the progression of fermentation and the development of cacao sensory profiles [57,58].

Sequencing of the 16S rRNA gene enabled the identification of bacteria belonging mainly to acetic acid bacteria (AAB) of the genus *Acetobacter*, represented by *Acetobacter pasteurianus*, *A*. *tropicalis*, *A*. *fabarum*, and *A*. *ghanensis*, as well as species of *Bacillus* (*B*. *subtilis* and *B*. *velezensis*), *Lysinibacillus phoenicis*, *Pseudomonas putida*, and *Staphylococcus epidermidis* (Table 2). Among the bacterial isolates, *A*. *pasteurianus* was recovered at a higher frequency than the other bacterial taxa during several fermentation stages, particularly from 24 to 120 h, whereas *B*. *subtilis* was also repeatedly isolated. *P*. *putida* and *S*. *epidermidis* were detected only at 144 h. Because these observations were obtained using culture-dependent isolation, the frequency of recovery should not be interpreted as a direct measure of taxon abundance or community dominance [46,54].

The higher recovery frequency of isolates belonging to the genus *Acetobacter* is consistent with studies recognizing these microorganisms as the dominant bacteria during the intermediate and final stages of cacao fermentation because of their ability to oxidize the ethanol produced by yeasts and convert it into acetic acid, thereby promoting the biochemical changes responsible for the development of bean quality [51,53]. The recovery of *Bacillus subtilis* and *B*. *velezensis* at intermediate and late fermentation stages indicates that these taxa were culturable under the conditions prevailing at those sampling points. However, their physiological persistence or ecological dominance cannot be established from the culture-dependent approach used here. In contrast, the sporadic detection of *Pseudomonas putida* and *Staphylococcus epidermidis* likely corresponds to environmental microorganisms introduced during process handling rather than members of the more frequently recovered fermentative microbiota [54,58,59].

#### 3.2.1. Temporal Dynamics of the Microbial Community During Spontaneous Cacao Fermentation

The temporal distribution of culturable microorganisms (Figure 6a) showed that yeasts and acetic acid bacteria (AAB) were recovered at several fermentation stages, whereas spore-forming bacteria were recovered more frequently from 72 h onward, and other bacterial groups were detected only at 144 h. This pattern reflects changes in the frequency and timing of recovery of culturable microorganisms under the experimental conditions rather than quantitative changes in microbial abundance within the fermentation system [60,61].

The higher recovery frequency of culturable yeasts during the initial stages is consistent with the availability of fermentable sugars in cocoa pulp and the recognized capacity of yeasts to metabolize these substrates during the early stages of fermentation [62]. As fermentation progresses, changes in substrate availability and the physicochemical conditions of the fermenting mass have been associated with reduced yeast recovery and the detection of other microbial groups in cocoa fermentations [46,61].

In parallel, AAB, particularly members of the genus *Acetobacter*, are known to oxidize ethanol to acetic acid and may contribute to acidification and other biochemical transformations associated with cocoa fermentation [46,62]. In the present study, the recovery of AAB at several fermentation stages coincided temporally with the recovery of yeasts, but the culture-dependent design does not allow direct inference of metabolic interactions or causal relationships between these groups. Similarly, the higher recovery frequency of spore-forming bacteria from 72 h onward may be related to their capacity to tolerate stressful fermentation conditions, although temperature, nutrient availability, and other environmental variables were not directly monitored in this study [60].

#### 3.2.2. Temporal Distribution of Culturable Yeast Genera

Figure 6b showed that *Pichia* isolates were recovered at higher frequencies during the first 72 h of fermentation, whereas *Saccharomyces* isolates were recovered more frequently between 72 and 120 h, with the highest recovery frequency observed at 96 h. This temporal pattern indicates changes in the frequency and timing of recovery of culturable yeast genera during spontaneous fermentation [63,64]. However, these culture-dependent observations cannot be interpreted as quantitative changes in the total yeast community.

The higher recovery frequency of *Pichia* isolates during the initial stages is consistent with the reported ability of some *Pichia* species to utilize fermentable sugars and produce extracellular enzymes under fermentation conditions [65]. The pectinolytic screening performed in the present study also indicates that some isolates exhibited a detectable capacity to degrade pectin in vitro; however, this qualitative response cannot be directly extrapolated to pectin degradation during cacao fermentation.

As fermentation progressed, the higher recovery frequency of *Saccharomyces* between 72 and 120 h coincided temporally with a shift in the culturable yeast genera recovered from the fermentation system. Members of *Saccharomyces* are well recognized for their tolerance to ethanol and other fermentation-related stresses and for their ability to produce ethanol and volatile metabolites relevant to cocoa flavor development [63,66]. Nevertheless, because temperature, sugar depletion, and ethanol accumulation were not directly monitored in the present study, their contribution to the temporal recovery pattern of *Saccharomyces* cannot be established.

#### 3.2.3. Temporal Distribution of Culturable Bacterial Genera

Figure 6c showed that *Acetobacter* was the most frequently recovered bacterial genus across the sampled fermentation stages, whereas *Bacillus* was recovered more frequently from 72 h onward and remained detectable until 144 h. *Lysinibacillus*, *Pseudomonas*, and *Staphylococcus* were recovered sporadically, mainly during the final stages of fermentation. Overall, these results indicate temporal shifts in the recovery frequency of culturable bacterial genera throughout spontaneous cacao fermentation [11,60].

The frequent recovery of *Acetobacter* is consistent with the established role of this genus in cocoa fermentation, particularly its capacity to oxidize ethanol to acetic acid under aerobic conditions [67,68]. This metabolic potential may contribute to acidification and to the physicochemical changes that characterize the later stages of fermentation. The higher recovery frequency of *Bacillus* from 72 h onward may reflect the ability of members of this genus to tolerate the increasingly stressful conditions associated with cocoa fermentation and to produce extracellular hydrolytic enzymes, including proteases and pectinolytic enzymes [40]. These enzymatic capabilities have been associated with the degradation of cocoa pulp and the transformation of macromolecular substrates, although their contribution to these processes was not directly quantified in the present study. In contrast, the sporadic recovery of *Lysinibacillus*, *Pseudomonas*, and *Staphylococcus* during the final stages may reflect their occurrence at low recoverable frequencies or their emergence under changing fermentation conditions, including increased environmental exposure during bean turning [60].

### 3.3. Analysis of Volatile Compounds

Cluster analysis (Figure 7a) identified four groups with distinct chemical profiles during cacao fermentation. Cluster 3 mainly comprised yeast samples collected at 0, 24, and 48 h, together with the testa sample collected at 0 h, corresponding to the early fermentation stages. Cluster 2 grouped the cacao bean samples collected between 0 and 120 h, whereas Cluster 1 included most bacterial samples and the bean and testa samples from the final fermentation stages (96–144 h). Finally, Cluster 4 was represented exclusively by the bacterial sample collected at 144 h, indicating a distinct volatile profile relative to the other clusters.

During the first 48 h, *Pichia kudriavzevii*, *Saccharomyces cerevisiae*, and *Hanseniaspora opuntiae* were recovered at relatively high frequencies, coinciding temporally with the detection of alcohols and esters [69]. The subsequent recovery of *Acetobacter pasteurianus* coincided with further changes in the volatile profile [70]. These temporal associations are consistent with the reported metabolic capacities of yeasts and acetic acid bacteria and provide a plausible biochemical context for the observed volatile patterns [71].

The mean values for each cluster (Figure 7b) revealed differences in the abundance of volatile compound families. Cluster 3 exhibited a higher relative representation of alcohols and esters; Cluster 2 was characterized by aldehydes and ketones; Cluster 1 showed a higher relative representation of acids; and Cluster 4 was characterized mainly by compounds grouped as “Others”.

The distribution of these compound families was consistent with the metabolic progression reported in previous studies on cacao fermentation, although the environmental parameters (pH, temperature, sugar content) underlying that progression were not directly measured in the present trial. Alcohols and esters showed relatively high representation during stages associated with greater recovery frequencies of culturable yeasts, whereas acids were more prominent during later stages [69]. The higher relative representation of compounds grouped as “Others” in Cluster 4 may reflect the accumulation or persistence of minor volatile constituents during the later stages of fermentation [3,70].

Principal component analysis (Figure 7c) showed that Dimension 1 explained 38.6% of the variability and Dimension 2 explained 24.1%, together explaining 62.7% of the total variance. Alcohols were associated with negative values of Dimension 1, whereas acids and compounds grouped as “Others” showed a positive association. Esters were mainly associated with the positive axis of Dimension 2.

The distribution of the variables indicates that the relative representation of volatile compound families differed among fermentation stages. Alcohols were associated with the early stages in which culturable yeasts were recovered more frequently, whereas acids were more strongly associated with later stages characterized by the recovery of *Acetobacter* and other oxidative bacteria [70]. Esters were located close to the origin of the factorial plane, suggesting a less pronounced association with either principal dimension and potentially reflecting their occurrence across multiple fermentation stages [72,73].

The biplot (Figure 7d) showed a temporal separation of the samples according to fermentation stage. Early stages were mainly associated with alcohols, intermediate stages with esters, terpenes, and phenols, whereas bacterial samples collected at 144 h were associated with compounds classified as “Others”. These patterns indicate that fermentation stage was associated with changes in the volatile compound profile. Overall, the temporal correspondence between culturable microbial recovery and volatile profiles supports an association between microbial dynamics and chemical changes during fermentation but does not establish taxon-specific causality.

The early recovery of culturable yeasts coincided with a greater relative representation of alcohols and esters, whereas later recovery of acetic acid bacteria coincided with a greater representation of acids and other volatile constituents. These patterns are consistent with the metabolic activities reported for these microbial groups during cacao fermentation [3,74].

#### 3.3.1. Alcohols

Across all three matrices, alcohols showed their highest relative abundance during the early fermentation stages (0–48 h) (Figure 8a–c). However, the timing of maximum abundance differed among matrices. In the beans, alcohol abundance was already high at 0 h (~33%) and reached its maximum at 48 h (~45%). It then decreased progressively to approximately 17% at 144 h. This early-to-intermediate maximum coincided with the highest recovery frequencies of culturable yeasts. This pattern is consistent with the known ability of yeasts to convert fermentable sugars into ethanol and higher alcohols [63]. These metabolites can subsequently contribute to the formation of aroma precursors. The later decline has been associated in previous studies with sugar depletion and ethanol oxidation by acetic acid bacteria [75]. However, sugar and ethanol contents were not directly measured in the present study.

In the testa, alcohols showed their highest relative abundance at the beginning of fermentation (~40% at 0 h). Their abundance decreased progressively thereafter. This pattern is consistent with the close physical contact between the testa and the fermenting mucilage. Such contact may facilitate the retention or exchange of volatile metabolites during the early fermentation stages [76]. The subsequent decrease may also be related to volatilization and transformation of these compounds as fermentation progresses [63].

In the microbial samples, alcohols were essentially undetectable at 0 h but increased sharply to 62% at 24 h. They reached approximately 83% at 48 h, the highest relative abundance recorded in the dataset. This pattern is consistent with high yeast metabolic activity associated with ethanol and higher-alcohol production during this period [75]. Alcohol levels subsequently declined during the bacteria-dominated stages (72–144 h). This decline is consistent with the reported use of alcohols by acetic acid bacteria as substrates for acetic acid production [63]. Maximum alcohol abundance occurred at 0 h in the testa and at 48 h in the beans and microbial samples.

#### 3.3.2. Esters

Esters showed contrasting temporal patterns among the three matrices (Figure 8a–c). In the beans, ester abundance increased progressively during fermentation. Esters were nearly undetectable at 0 h and reached their highest relative abundance at 120 h (~27%). These compounds are commonly associated with fruity and floral aroma notes in cacao [77]. The observed increase is consistent with the metabolic activity of yeasts, which can synthesize esters from alcohols and organic acids during alcoholic fermentation [62]. Similarly, Ooi et al. [57] associated increasing ester levels with the fermentative activity of Saccharomyces and Pichia.

In contrast, esters in the testa were relatively abundant during the early and intermediate stages of fermentation (~30–35% between 0 and 96 h). Their relative abundance decreased toward the end of the process, reaching ~17% at 144 h. This pattern is consistent with the early transfer of volatile metabolites from the fermenting mucilage to the testa [57,62]. The subsequent decrease may reflect changes in ester formation, retention, or redistribution as fermentation progressed.

Esters in the microbial samples also showed higher relative abundance during the early stages. They reached their maximum at 0 h (~48%) and remained relatively abundant during the stages characterized by greater yeast recovery. Their abundance subsequently declined and reached its lowest value at 144 h (~1%). This pattern is consistent with the reported association between yeast metabolism and ester formation during alcoholic fermentation [57]. Esters are important secondary metabolites because they contribute to desirable sensory characteristics and may also reflect changes occurring during fermentation [77]. The contrasting trajectories among matrices suggest differences in the temporal distribution of esters during fermentation. Their relative abundance increased in the beans but generally decreased in the testa and microbial samples. This pattern may reflect differences in ester formation, retention, and transfer among the analyzed matrices rather than synchronous changes throughout the fermentation system.

#### 3.3.3. Acids

Acids increased progressively across all three matrices as fermentation advanced (Figure 8a–c). In the beans, this increase is consistent with the diffusion of organic acids from the mucilage into the bean [73]. A similar progressive increase in organic acids during cacao fermentation was reported by Sari et al. [78].

In the testa, the increase may be related to direct contact with the fermenting mucilage and the transfer of metabolites produced by the culturable microbiota [79]. Delgado-Ospina et al. [80] reported a similar pattern and associated it with biochemical changes involved in the development of cacao sensory quality. In the microbial samples, the higher relative abundance of acids is consistent with the metabolic activity of acetic acid bacteria. These microorganisms oxidize ethanol produced by yeasts to form acetic acid [10]. Similar metabolic patterns during cacao fermentation have been reported by Cruz-Rojas et al. [81].

Across the three matrices, the progressive increase in acids is consistent with processes previously described during cacao fermentation, particularly ethanol oxidation by acetic acid bacteria [10,81]. This process can lead to acetic acid accumulation, a decrease in pH, and physicochemical changes in the fermenting mass. However, pH, ethanol content, and embryo viability were not directly measured in the present study. Therefore, these mechanisms should be considered literature-based explanations rather than causal relationships demonstrated by the present experiment.

#### 3.3.4. Aldehydes

Aldehydes showed divergent temporal patterns among the three matrices (Figure 8a–c). In the beans, aldehyde abundance was highest during the earliest stages of fermentation (~22–35% between 0 h/pod and 0 h). It then decreased progressively and reached its lowest value at 144 h (~5%). This high initial abundance may reflect aldehydes already present in, or rapidly formed from, unfermented pod and bean tissues. This pattern differs from the accumulation associated with amino acid degradation and lipid oxidation after embryo death reported in other cacao fermentation systems [70,71]. However, these biochemical processes were not directly evaluated in the present study.

In the testa, aldehydes increased from a low initial value (~3%) to a maximum at 120 h (~12%). Their relative abundance then decreased sharply to approximately 3% at 144 h. This pattern is broadly consistent with metabolite transfer during fermentation and oxidative reactions in the outer bean tissues [70,82]. However, the decline at 144 h indicates that aldehyde accumulation in the testa was not sustained during the final stage of fermentation. In the microbial samples, aldehydes remained at low and comparatively stable levels throughout the process, with no clear temporal trend. This pattern suggests no strong association with a specific fermentation stage under the conditions evaluated [71,82].

#### 3.3.5. Ketones

Ketones showed distinct temporal patterns across the three matrices (Figure 8a–c). In the beans, ketone abundance fluctuated throughout fermentation (~8–16%) and reached its highest value at 144 h (~20%). This pattern is consistent with lipid oxidation and other biochemical transformations reported in the cotyledon after embryo death [83]. Ketones have also been associated with characteristic aromatic notes in cacao, as reported by Chóez-Guaranda et al. [84]. However, embryo viability and lipid oxidative status were not directly assessed in the present study.

In the testa, ketones remained relatively stable (~8–15%) during most of the fermentation process. Their relative abundance decreased sharply at 144 h to approximately 4%. This pattern only partially agrees with the expected increase in ketone formation during intermediate and late fermentation stages [81]. The marked decline at 144 h indicates that ketone abundance did not continue to increase toward the end of fermentation.

In the microbial samples, ketones were essentially undetectable during the early stages associated with greater yeast recovery (0–48 h). They were first detected at 72 h and reached their maximum at 96 h (~33%). Their relative abundance then declined slightly toward 144 h. This temporal pattern coincided with stages characterized by greater bacterial recovery rather than with the early yeast-associated stages [70,83]. Ketone dynamics differed among matrices. The beans showed a fluctuating increase, the testa showed a relatively stable pattern followed by a late decline, and the microbial samples showed a later increase during bacteria-associated stages.

#### 3.3.6. Terpenes and Hydrocarbons

Terpenes and hydrocarbons showed a broadly similar temporal pattern in the beans and microbial samples, in contrast to the testa (Figure 8a–c). In the beans, both compound classes were most abundant at the pre-fermentation stage (0 h/pod, ~20% terpenes and ~9% hydrocarbons). Their relative abundance declined sharply thereafter and remained low and comparatively stable throughout the rest of the fermentation period (0–144 h). This early decline is consistent with the transformation of endogenous bean metabolites and the release of aromatic compounds into the cotyledon as fermentation begins [82]. The residual terpene and hydrocarbon fractions may also contribute to the floral, herbal, and resinous notes of cacao [81].

In the microbial samples, hydrocarbons showed a similar pattern and were detected only transiently at 0 h (an early stage associated with yeast recovery, ~10%). They remained essentially undetectable thereafter, whereas terpenes were negligible throughout the fermentation process. This transient occurrence during the early stage is consistent with an indirect relationship with fermentative activity. Previous studies have reported that microbial activity can promote the transformation and release of aroma precursors during the initial stages of fermentation [69]. This observation is also consistent with Tejeda et al. [70], who reported that microbial dynamics influence the final volatile composition and, consequently, the sensory quality of cocoa.

The testa, by contrast, showed a more variable trajectory. Terpene abundance decreased after 0 h, increased to a maximum between 48 and 72 h (~17–18%), and then declined to ~2% at 144 h. This fluctuation may reflect interactions between compounds present in the external tissues of the bean and metabolites generated during fermentation [70]. Erazo Solorzano et al. [82] similarly reported changes in these compounds as fermentation and drying progressed.

Terpenes and hydrocarbons showed an early and transient pattern in the beans and microbial samples. In the testa, however, they persisted longer and reached their highest relative abundance during the intermediate stages of fermentation.

#### 3.3.7. Other Compounds

Compounds classified as “Others” primarily comprised lactones and sulfur- and nitrogen-containing compounds. Their temporal patterns differed markedly among the three matrices (Figure 8a–c). In the cocoa beans, their relative abundance remained low at the beginning of fermentation (~3% at 0 h/pod and 0 h). It generally increased, despite some fluctuations, to ~7–11% between 72 and 144 h. This pattern may reflect the progressive formation, transformation, or accumulation of minor metabolites during fermentation. Although present at lower relative abundances than major families such as alcohols, acids, and esters, these compounds may contribute to overall aroma complexity and provide complementary information on fermentation-associated changes [71,85].

In the testa, compounds grouped as “Others” showed a broadly similar increase from low initial levels (~0% at 0 h) to ~8–10% between 96 and 144 h. This pattern may be associated with metabolite exchange among bean tissues and the accumulation or transformation of minor volatile compounds as fermentation progressed [71,86].

In the microbial samples, this family was essentially undetectable from 0 to 120 h but showed a marked increase at 144 h. At this time point, it accounted for approximately 75% of the relative VOC profile. This unusual increase was associated primarily with 1,2-dimethylhydrazine, a nitrogen-containing compound tentatively identified in the selected microbial culture analyzed at 144 h. Given its abrupt detection at this single time point, its origin cannot be established from the present dataset. The signal could reflect isolate-specific metabolism under the culture conditions, contamination or contributions from the culture medium, or uncertainty associated with the tentative GC-MS identification. Although changes in nitrogen-containing compounds are known to occur during cocoa fermentation [87], the present analysis does not provide sufficient evidence to directly link the detected compound to nitrogen metabolism in the fermenting cocoa mass. Confirmation would require analysis of uninoculated medium controls and, ideally, compound identification using an authentic standard.

#### 3.3.8. Dynamics of the Major Compounds During the Spontaneous Fermentation of Criollo Cacao

The relative abundance of esters in the cacao beans (Figure 9) increased progressively throughout fermentation, reaching its highest value at 120 h and its lowest value at 0 h (Table S1).

Among the predominant compounds, 1-butanol, 3-methyl-, acetate increased from 24 h onward, with greater abundance during the intermediate and final stages, whereas 2-heptanol, acetate showed the highest relative abundance between 120 and 144 h. Likewise, acetic acid, 2-phenylethyl ester was present throughout most of the process, reaching its highest abundance at 144 h, whereas 2-pentanol, acetate progressively increased until 120 h. This temporal pattern is consistent with the progressive redistribution and diffusion of volatile esters into the cocoa beans during fermentation, as reported in previous studies [72,88]. Such changes may be facilitated by physicochemical modifications of the bean matrix occurring during fermentation.

This temporal pattern coincided with changes in the frequency of recovery of culturable yeasts and bacteria during fermentation. *Pichia kudriavzevii*, *Saccharomyces cerevisiae*, and *Hanseniaspora opuntiae* were recovered at different fermentation stages, and their reported metabolic capacities are consistent with the production of higher alcohols and esters through yeast-mediated transformations [58,75]. Likewise, *Acetobacter pasteurianus* was recovered at several fermentation stages, particularly during the later stages. Its reported ability to oxidize ethanol and contribute to organic acid formation may have influenced the ester profile indirectly by modifying the pool of acid precursors. However, these temporal associations should not be interpreted as direct evidence that specific microbial taxa were responsible for the observed changes in ester relative abundance, because the microorganisms were characterized using a culture-dependent approach.

Esters in the testa (Figure 9b) showed their highest abundance at the beginning of fermentation (0 h), followed by a progressive decrease until 144 h. 1-Butanol, 3-methyl-, acetate was detected throughout the process, with greater abundance between 96 and 120 h, whereas decanoic acid, ethyl ester predominated between 0 and 48 h and decreased as fermentation progressed. Likewise, dodecanoic acid, ethyl ester was more abundant at 48 and 144 h, whereas ethyl 2-(5-methyl-5-vinyltetrahydrofuran-2-yl)propan-2-yl carbonate, 2,4-diacetoxypentane, 2-octanol, acetate, 2-nonanol, acetate, and ethyl 9-decenoate were more abundant during the initial hours and subsequently decreased. A similar pattern has been reported during cacao fermentation, in which ester composition changes as temporal changes in the recovery frequency of culturable microorganisms progresses and metabolites diffuse into the bean tissues [72,88].

This behavior is consistent with the intense initial activity of yeasts present in the mucilage and on the bean surface, mainly *Pichia kudriavzevii*, *Saccharomyces cerevisiae*, and *Hanseniaspora opuntiae*, which are responsible for the production of volatile alcohols and esters. Subsequently, the increase in acetic acid bacteria and the depletion of fermentable sugars reduce the synthesis of these compounds, thereby promoting changes in the aroma profile throughout fermentation [56,89].

Esters detected in the microbial samples (Figure 9c) were most abundant at 0 h and occurred at lower levels at 48 and 144 h. 1-Butanol, 3-methyl-, acetate predominated during the initial stage (0–24 h), whereas 1-butanol, 2-methyl-, acetate, octanoic acid, ethyl ester, and decanoic acid, ethyl ester also showed greater abundance during the first hours of fermentation, coinciding temporally with the higher frequency of recovery of yeast isolates during the early stages [72]. In contrast, ethyl acetate increased in abundance between 72 and 120 h, reaching its maximum at 96 h, when the population of acetic acid bacteria increased.

These results demonstrate the characteristic metabolic transition of cocoa fermentation, in which yeasts initially produce higher alcohols that serve as substrates for ester synthesis, whereas the reported metabolic potential of *Acetobacter pasteurianus* includes ethanol oxidation, which may contribute to changes in the volatile compound profile through the formation of acetic acid and other metabolites [75,90].

Alcohols in the cocoa beans (Figure 10a) exhibited a variable distribution throughout fermentation. 1-Butanol, 3-methyl- showed low abundance at the beginning of fermentation. Its relative abundance increased between 24 and 48 h and then progressively declined until 144 h. (R)-(-)-2-Pentanol was present throughout the process, although its abundance decreased at 24 h. It increased again at 48 h and subsequently declined toward the end of fermentation. Linalool was detected at all stages, with its highest relative abundance at 0 h (Table S1) and lower levels thereafter.

2-Heptanol was detected between 24 and 120 h, with higher relative abundance at 48 and 120 h. In contrast, 1-butanol, 2-methyl- was detected throughout fermentation (0–144 h), reaching its maximum relative abundance at 48 h. Its abundance progressively decreased thereafter. Phenylethyl alcohol was detected only at 0 and 48 h, with higher relative abundance at 48 h. By contrast, 3-buten-2-ol, 2-methyl- was restricted to the initial stage and showed its highest relative abundance at 0 h.

The temporal occurrence of these alcohols coincided with the recovery of *Pichia kudriavzevii, Saccharomyces cerevisiae*, and *Hanseniaspora opuntiae*. Previous studies have shown that yeasts can produce higher alcohols through sugar and amino acid metabolism, including the Ehrlich pathway [56,72]. This metabolic capacity provides a plausible biochemical context for the temporal pattern observed here. However, the present data do not establish a direct causal relationship between the isolated yeast taxa and the observed volatile compounds.

Alcohols in the testa (Figure 10b) were present throughout fermentation, although their abundance varied depending on the fermentation stage. Linalool was detected throughout the fermentation process and showed its highest relative abundance at 0 h, followed by a progressive decrease. 1-Butanol, 3-methyl- was detected from 0 to 144 h, with greater abundance up to 48 h and a progressive decrease toward the end of fermentation. (R)-(-)-2-Pentanol was also present throughout fermentation, reaching its maximum abundance at 144 h. 2-Heptanol was detected between 0 and 48 h and predominated at the beginning of the process, whereas 3-buten-2-ol, 2-methyl- was detected only at 0 and 48 h, with greater abundance at 0 h. Phenylethyl alcohol was detected exclusively at 0 h and showed high abundance.

The high relative abundance of (R)-(-)-2-pentanol in the testa at 144 h is noteworthy. At this stage, yeast recovery had declined, whereas AAB were more frequently recovered. This pattern is notable because (R)-(-)-2-pentanol is typically associated with yeast metabolism. Several mechanisms could potentially explain its late occurrence in the testa. One possibility is delayed transfer of (R)-(-)-2-pentanol produced earlier by yeasts from the pulp or bean matrix to the testa. Another possibility is residual yeast metabolic activity beyond the period of highest yeast recovery. A contribution from AAB-mediated metabolic transformations cannot be excluded. However, the present dataset does not allow these mechanisms to be distinguished. Targeted kinetic sampling or isotope-labeling experiments would be required to evaluate these possibilities. Therefore, they should be considered hypotheses for future investigation rather than conclusions of the present study.

The relatively high abundance of these alcohols reflects the intense initial fermentative activity of yeasts present in the mucilage, mainly *Pichia kudriavzevii* and *Saccharomyces cerevisiae*, which synthesize higher alcohols during alcoholic fermentation. As fermentable sugars are depleted and *Acetobacter* activity increases, these compounds are transformed or used as precursors for other aroma-related metabolites, progressively modifying the volatile profile of cacao [71,88].

Alcohols detected in the microbial samples (Figure 10c) exhibited temporal variation associated with the microbial dynamics of fermentation. 1-Butanol, 3-methyl- was detected at 24 and 48 h, reaching its highest abundance at 48 h. 1-Butanol, 2-methyl- was present throughout most of the fermentation process, except at 96 h, with its highest abundance at 0 h and a progressive decrease toward the end of fermentation. Phenylethyl alcohol was detected throughout the process, reaching its maximum abundance at 24 h. 2-Nonanol was present between 0 and 96 h, except at 24 h, and increased until reaching its highest abundance at 96 h. Likewise, 1-butanol predominated between 72 and 144 h, with its maximum abundance at 120 h, whereas 1-octen-3-ol was detected only at 48 h.

These results are consistent with the metabolic transitions commonly described during cacao fermentation, in which yeasts initially produce higher alcohols, whereas acetic acid bacteria subsequently modify these compounds through oxidative processes, thereby promoting the formation of new metabolites responsible for the aromatic complexity of fermented cacao [72,73].

Volatile compounds were identified by comparing their mass spectra with those in the NIST 17 Library database. Detailed chromatographic parameters (RT, RI, match factor, and relative abundance) corresponding to the beginning of fermentation (0 h) are presented in Table S1. Likewise, the temporal distributions of individual compounds belonging to the acid (Figures S5–S7), aldehyde (Figures S8–S10), ketone (Figures S11–S13), and terpene (Figures S14–S16) families in the cacao beans, testa, and microbial samples are presented in the Supplementary Materials. Acids reflect the progression of fermentation and bean acidification; aldehydes contribute to the generation of aromatic notes derived from amino acid and lipid metabolism; ketones originate from metabolic transformations and oxidative processes, contributing to the sensory profile; whereas terpenes, although less abundant, impart floral and herbal notes associated with cacao origin and aroma complexity [84,91].

### 3.4. Correlation Between Culturable Microorganisms and Volatile Compound Families

To further examine the temporal relationship between culturable microorganisms and volatile profiles, Spearman rank correlations were calculated between microbial recovery frequency and the relative abundance of 11 VOC families across the seven fermentation stages. Several strong associations were observed at the nominal significance level (*p* < 0.05). The highest positive association was observed between *Acetobacter pasteurianus* and the relative abundance of the “Others” VOC family (ρ = 0.927, *p* = 0.003), followed by positive associations of *Bacillus subtilis* with esters (ρ = 0.852, *p* = 0.015) and *A. pasteurianus* with phenols (ρ = 0.846, *p* = 0.016). *Pichia kudriavzevii* showed a positive association with alcohols (ρ = 0.842, *p* = 0.017) and a negative association with esters (ρ = −0.805, *p* = 0.029). *A. tropicalis* also showed strong negative associations with esters, acids, and the “Others” family (ρ = −0.802 for each), together with a positive association with hydrocarbons (ρ = 0.802) and alcohols (ρ = 0.757). These relationships are illustrated in Figure 11 and summarized in Table S2.

These exploratory correlation patterns suggest that changes in the recovery frequency of culturable microorganisms co-occurred with changes in the relative abundance of VOC families during fermentation. For example, *Pichia kudriavzevii* showed a positive correlation with alcohols and a negative correlation with esters. Although the former pattern is compatible with the known fermentative metabolism of yeasts, it does not demonstrate a direct metabolic contribution to these VOC families. Similarly, the correlation patterns observed for *Acetobacter* species coincided with their recovery at different fermentation stages. They also coincided with changes in alcohols, acids, and other volatile compound families.

However, these relationships should be interpreted as exploratory temporal correlations rather than evidence of direct microbial effects on specific VOC families. The present culture-dependent approach provides information on the recovery frequency of culturable isolates. It does not directly quantify microbial population abundance or metabolic activity. Therefore, the observed correlations represent co-occurring temporal patterns rather than demonstrated functional interactions between microorganisms and VOCs.

Importantly, none of the 132 microorganism–VOC correlations remained significant after Benjamini–Hochberg correction using a false discovery rate threshold of 0.05 (all adjusted *p*-values > 0.05). Therefore, the correlations were interpreted as exploratory and used to identify potential temporal patterns and candidate microorganism–VOC relationships for further investigation. They were not used to infer causal microbial effects. This interpretation is particularly appropriate because the three fermentation boxes originated from the same cocoa lot. The boxes were operated simultaneously as parallel fermentation units within a single fermentation trial. Accordingly, they were not considered independent biological replicates.

## 4. Conclusions

The spontaneous fermentation of Criollo cacao showed temporal variation in the recovery of culturable yeasts and bacteria and in the relative abundance of VOC families. Molecular identification detected *Pichia kudriavzevii*, *Saccharomyces cerevisiae*, and *Hanseniaspora opuntiae* among the frequently recovered yeasts, together with several *Acetobacter* species and other bacterial taxa. Pectinolytic screening showed that all evaluated isolates formed visible degradation halos, with differences among isolates indicating variation in apparent pectinolytic potential. The volatile profile also changed throughout fermentation, particularly for alcohols, esters, acids, aldehydes, ketones, and other VOC families.

The integration of microbial recovery, molecular identification, pectinolytic screening, and volatile profiling revealed several temporal associations between culturable microorganisms and VOC families. In particular, *Pichia kudriavzevii*, *Acetobacter pasteurianus*, *Acetobacter tropicalis*, and *Bacillus subtilis* showed nominal associations with selected VOC families. However, none remained statistically significant after correction for multiple comparisons. These patterns should therefore be interpreted as exploratory evidence of temporal co-variation rather than microbial causation. Overall, the study identified culturable microorganisms and VOC patterns that provide a basis for strain-level functional studies and controlled fermentation experiments aimed at evaluating their contribution to cacao aroma development.

## Figures and Tables

**Figure 1:**
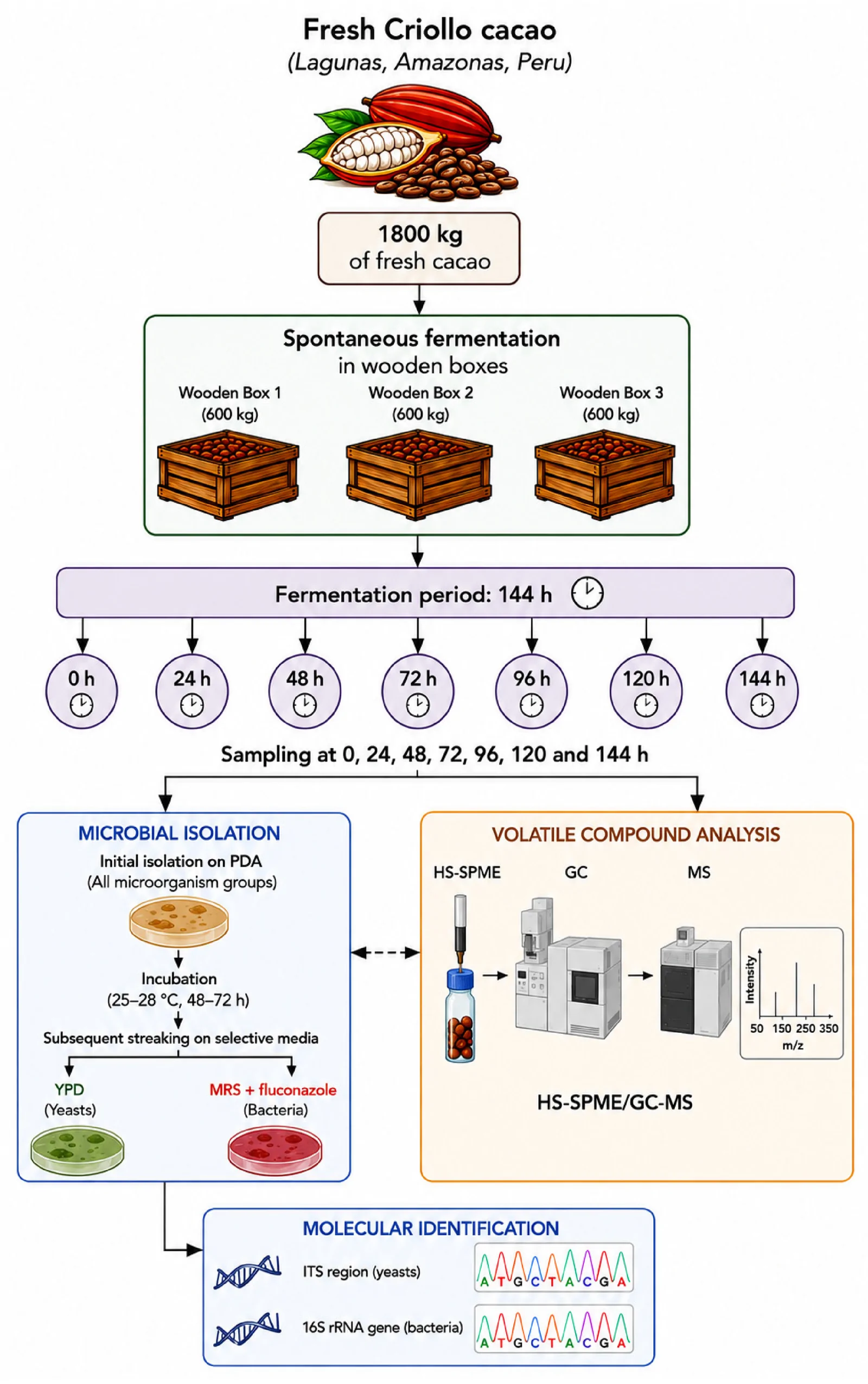
Schematic representation of the experimental design for the spontaneous fermentation, microbiological characterization, and volatile compound analysis of Criollo cacao.

**Figure 2:**
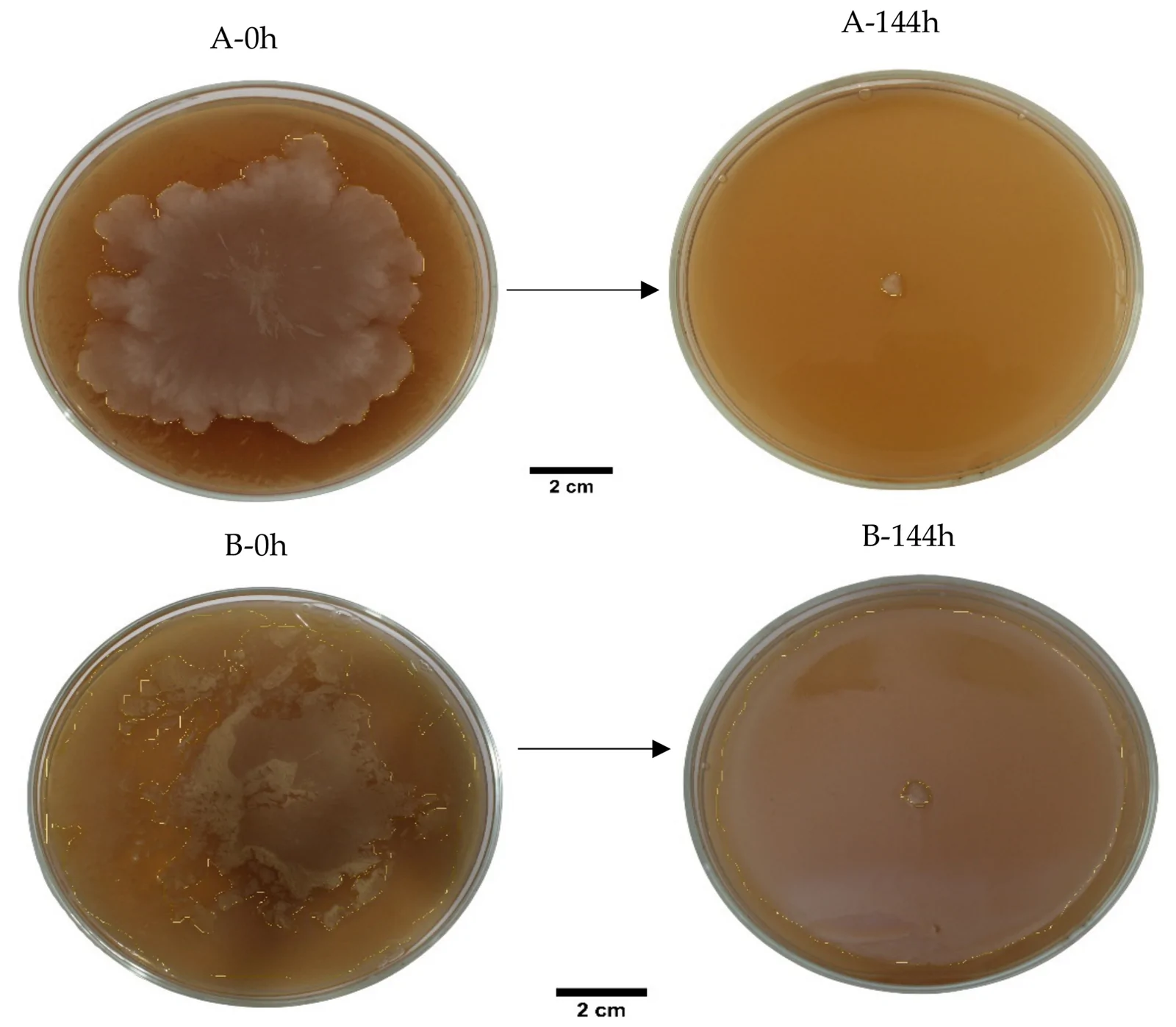
Morphology of representative yeast isolates recovered at 0 and 144 h of spontaneous Criollo cacao fermentation, before (**A**) and after (**B**) development of pectin degradation halos. Colony and halo diameter and area were measured using ImageJ v1.54p (Fiji distribution, National Institutes of Health, Bethesda, MD, USA).

**Figure 3:**
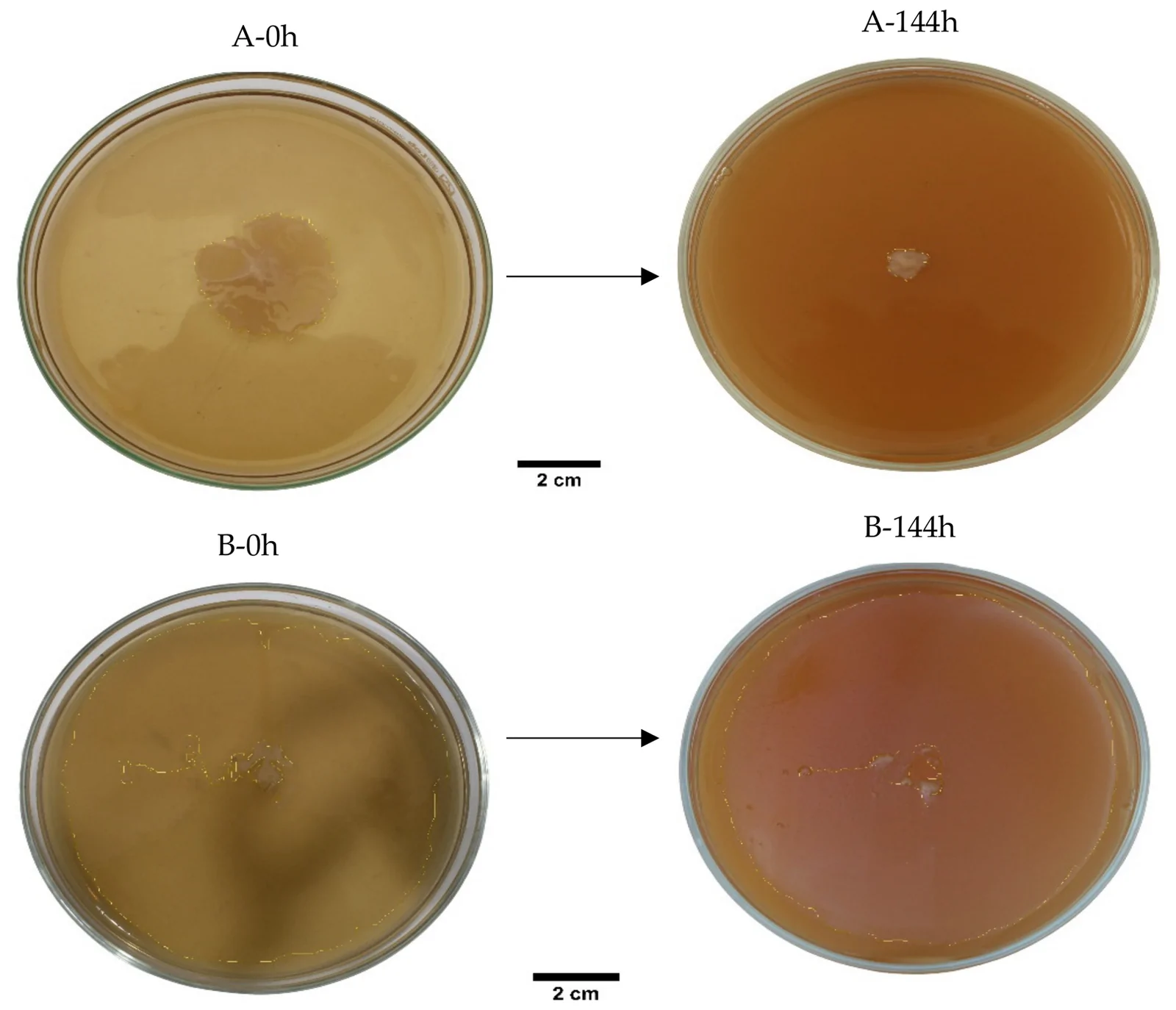
Morphology of representative bacterial isolates recovered at 0 and 144 h of spontaneous Criollo cacao fermentation, shown before (**A**) and after (**B**) development of pectin degradation halos, respectively. Colony and halo diameter and area were measured using ImageJ v1.54p (Fiji distribution, National Institutes of Health, Bethesda, MD, USA).

**Figure 4:**
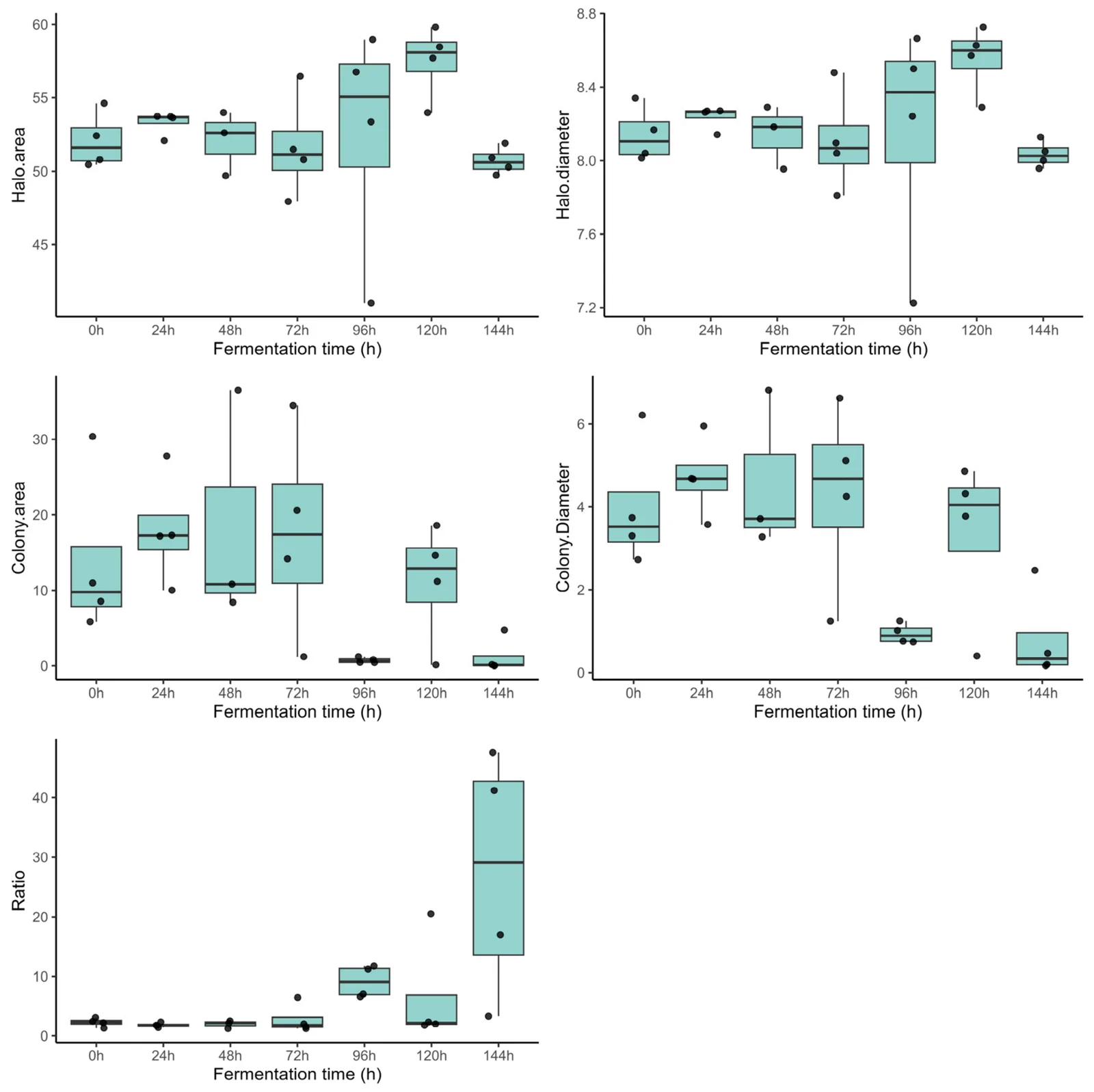
Box-and-whisker plot of the pectinolytic potential of yeasts isolated on YPD medium during the spontaneous fermentation of Criollo cacao from Lagunas, Cajaruro District, Utcubamba Province, Amazonas Region, Peru. Boxes represent the interquartile range (IQR) of halo/colony measurements, the horizontal line inside each box denotes the median, whiskers extend to 1.5× the IQR, and individual points beyond the whiskers denote outliers.

**Figure 5:**
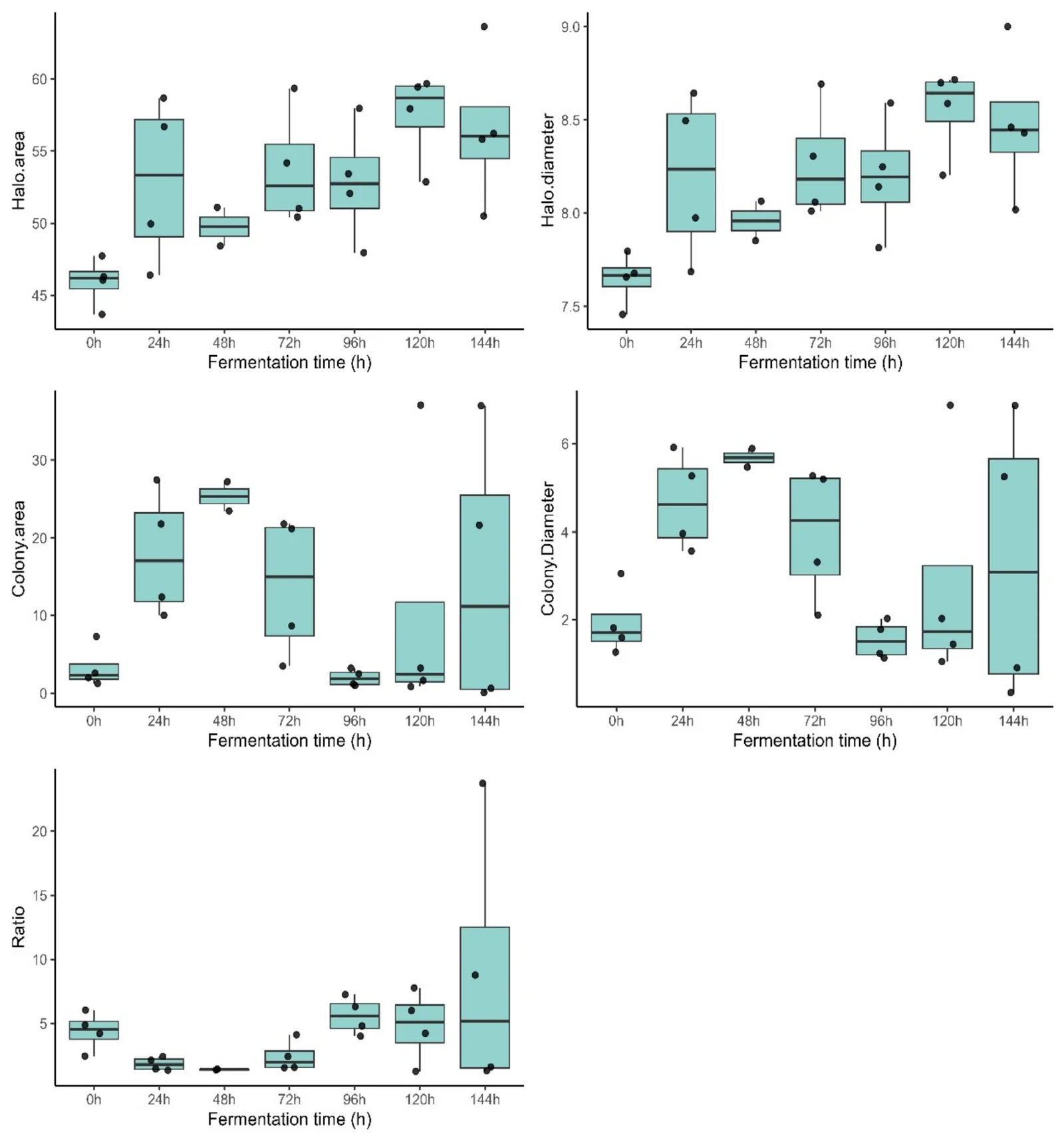
Box-and-whisker plot of the pectinolytic potential of bacterial isolates cultured on MRS medium during the spontaneous fermentation of Criollo cacao from Lagunas, Cajaruro District, Utcubamba Province, Amazonas Region, Peru. Boxes represent the interquartile range (IQR), the horizontal line inside each box denotes the median, whiskers extend to 1.5× the IQR, and individual points beyond the whiskers denote outliers.

**Figure 6:**
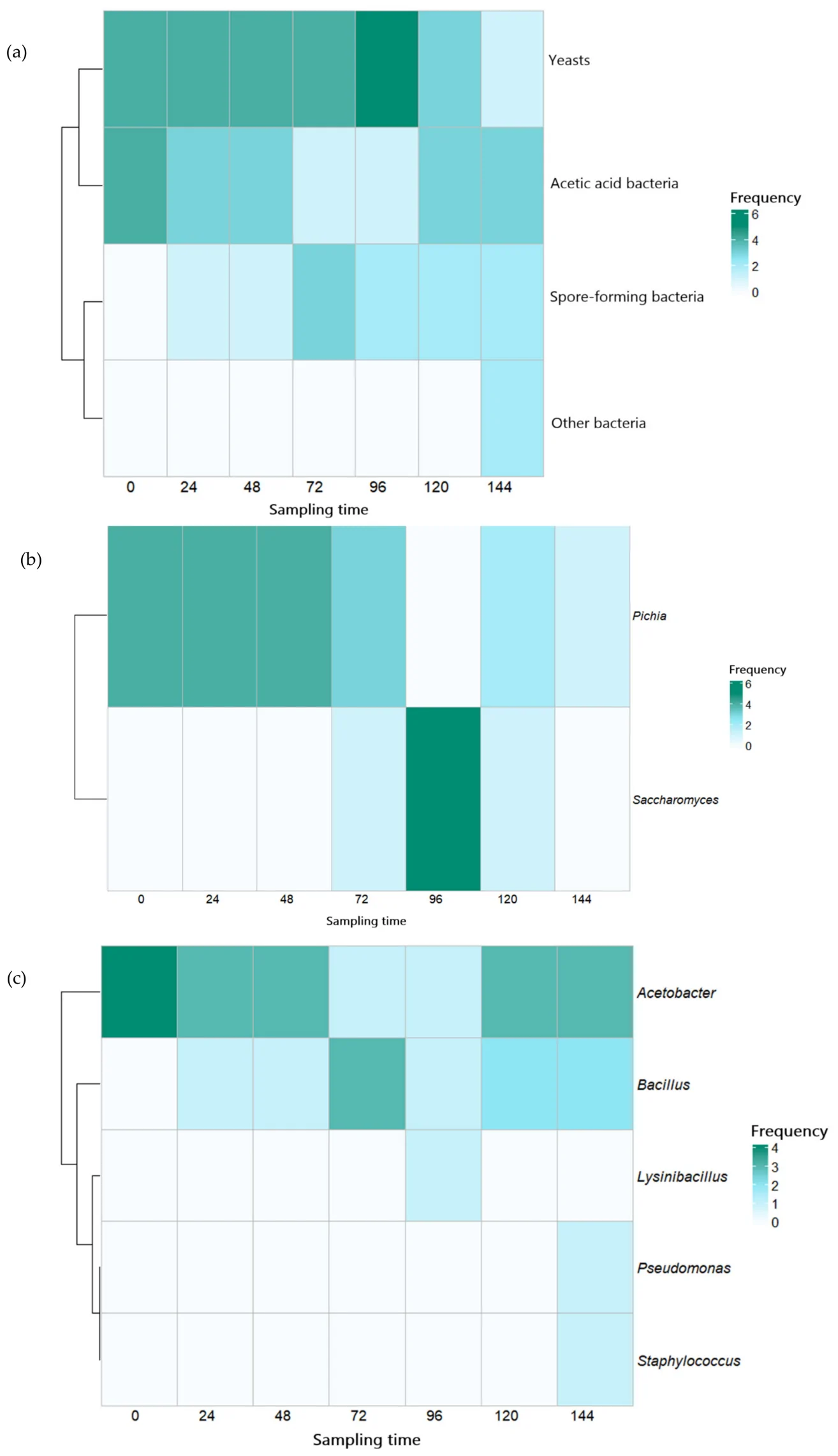
Temporal distribution of culturable microorganisms during spontaneous fermentation of Criollo cacao. (**a**) Main microbial groups, (**b**) fungal genera, and (**c**) bacterial genera recovered at 0, 24, 48, 72, 96, 120, and 144 h. Color intensity represents the relative frequency of recovery within the culture-dependent dataset. The figure reflects recovery patterns of culturable isolates and should not be interpreted as quantitative changes in total microbial abundance.

**Figure 7:**
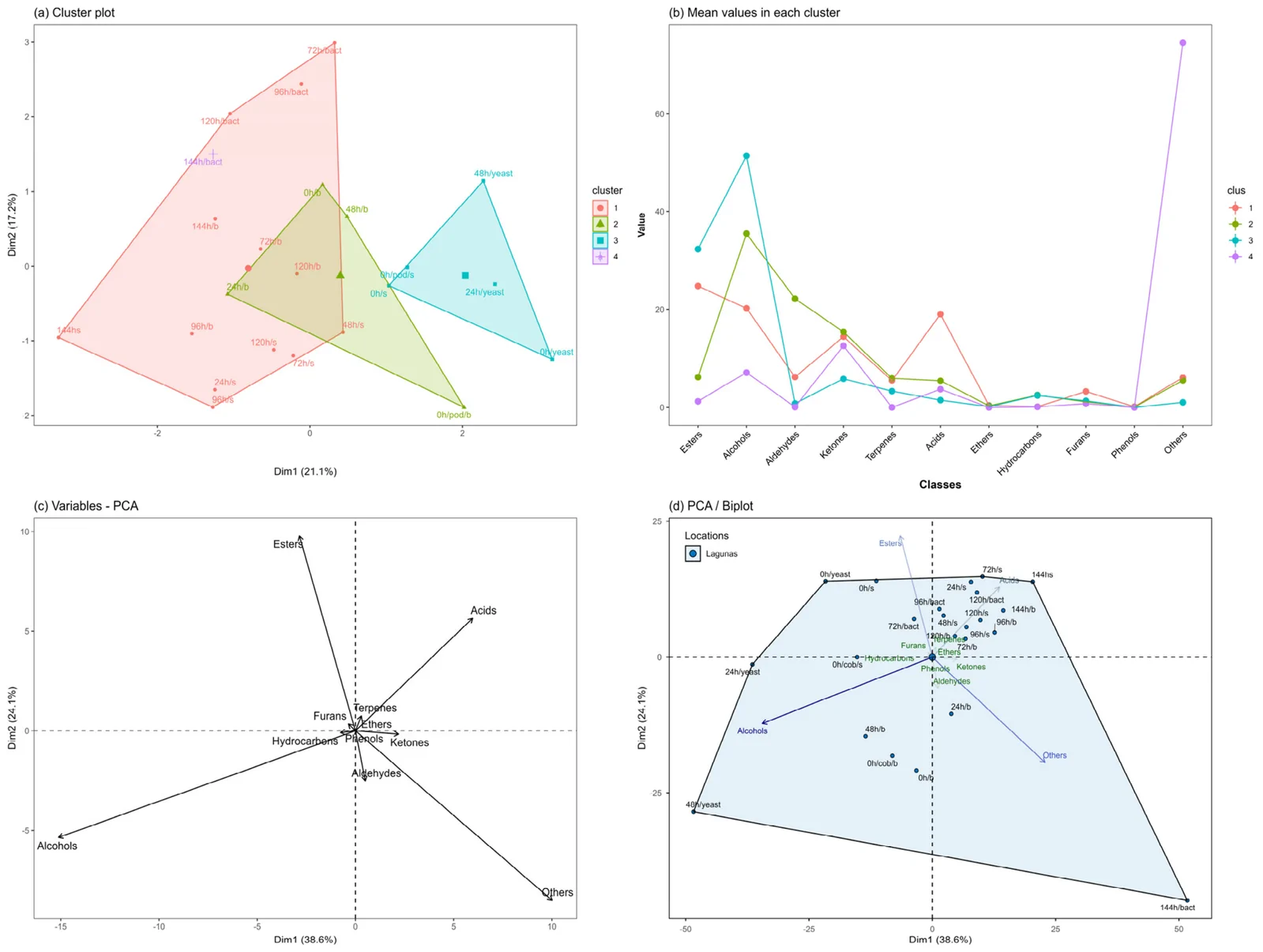
Multivariate characterization of volatile compound profiles during spontaneous fermentation of Criollo cacao: (**a**) cluster plots, (**b**) mean values in each cluster, (**c**) variables—PCA (Principal Component Analysis), and (**d**) PCA/biplot of the abundance of volatile compounds in the cocoa bean kernel, cocoa shell, and microbial matrices. Samples are grouped according to fermentation stage and matrix. VOC abundances are expressed as relative values because authentic standards and compound-specific calibration curves were not used.

**Figure 8:**
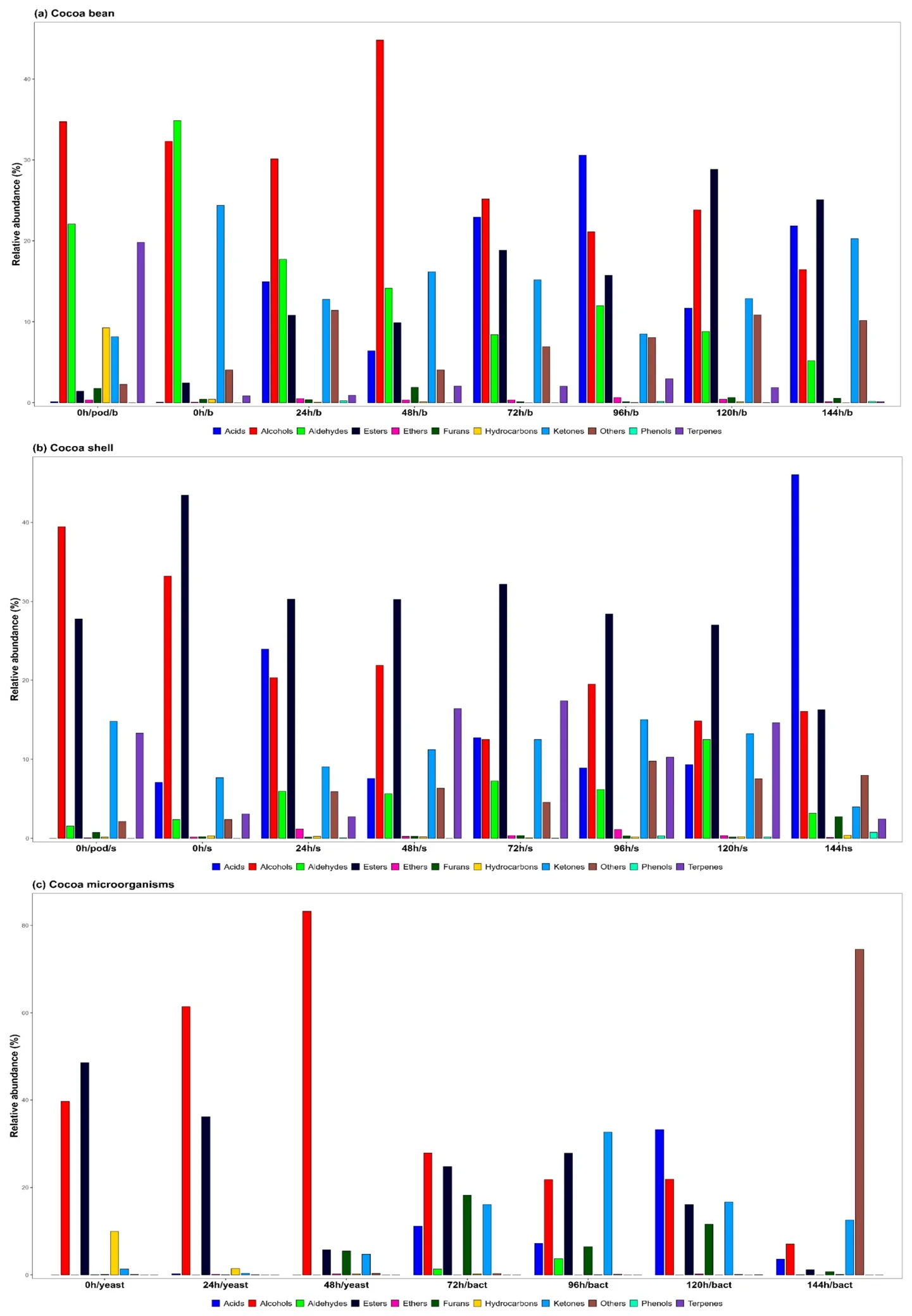
Families of volatile compounds detected at different stages of spontaneous fermentation of Criollo cocoa: (**a**) relative abundance in cocoa beans, (**b**) relative abundance in cocoa bean shell, and (**c**) relative abundance in isolated microorganisms (yeasts and bacteria).

**Figure 9:**
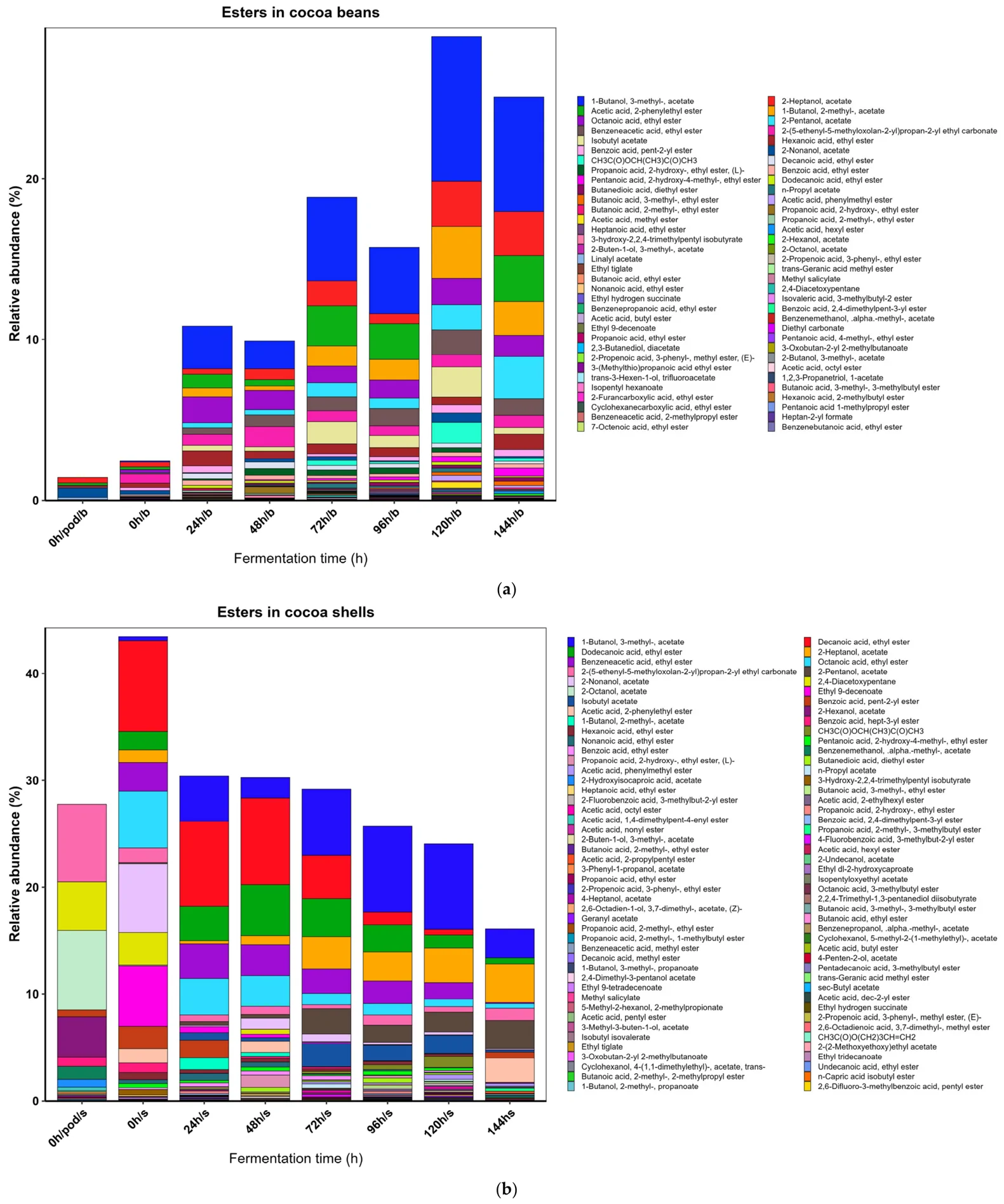

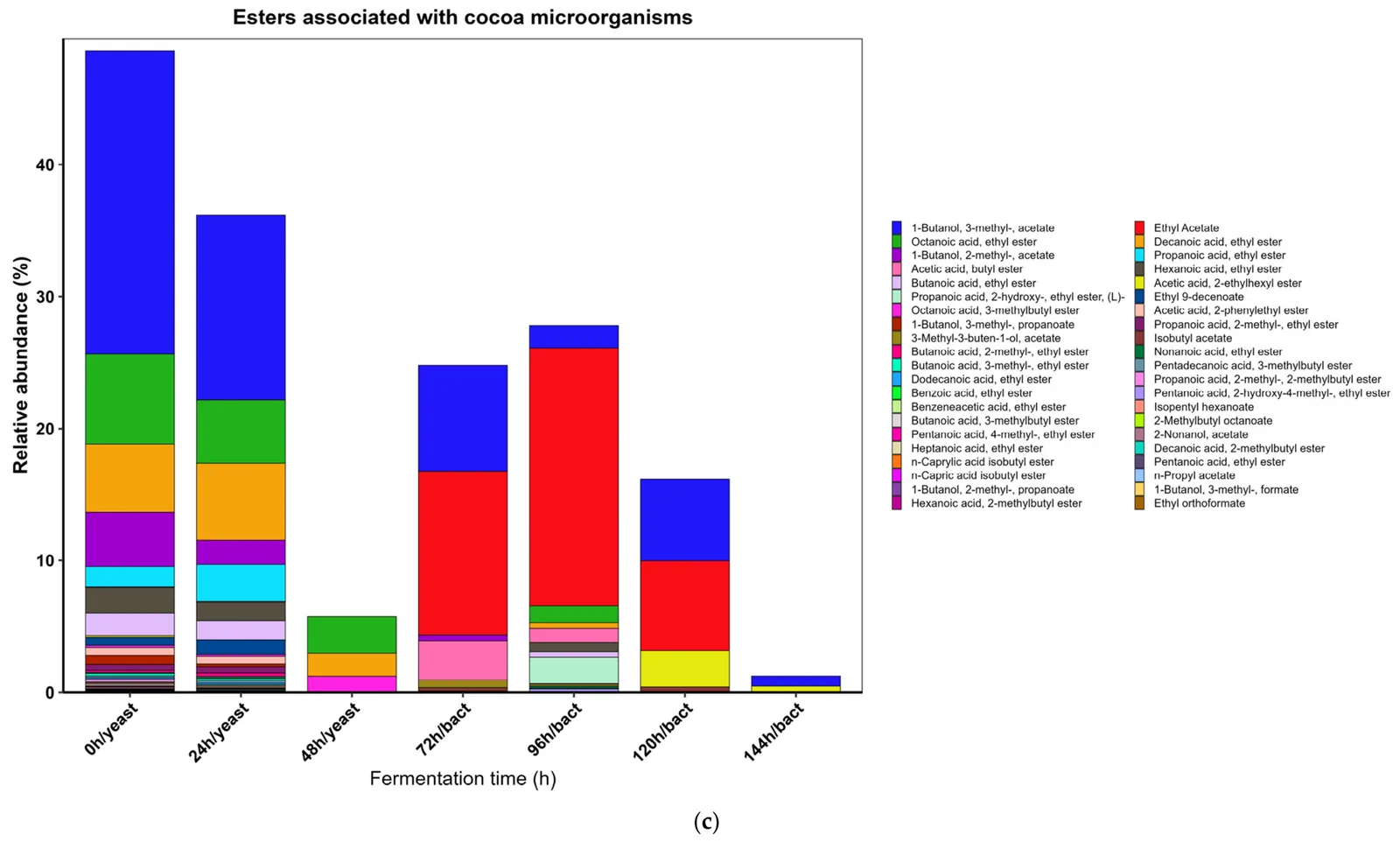
Stacked bar charts showing the relative abundance of volatile compounds within the ester family during spontaneous fermentation: (**a**) in the cocoa bean kernel, (**b**) in the cocoa bean shell, and (**c**) associated with isolated cocoa microorganisms.

**Figure 10:**
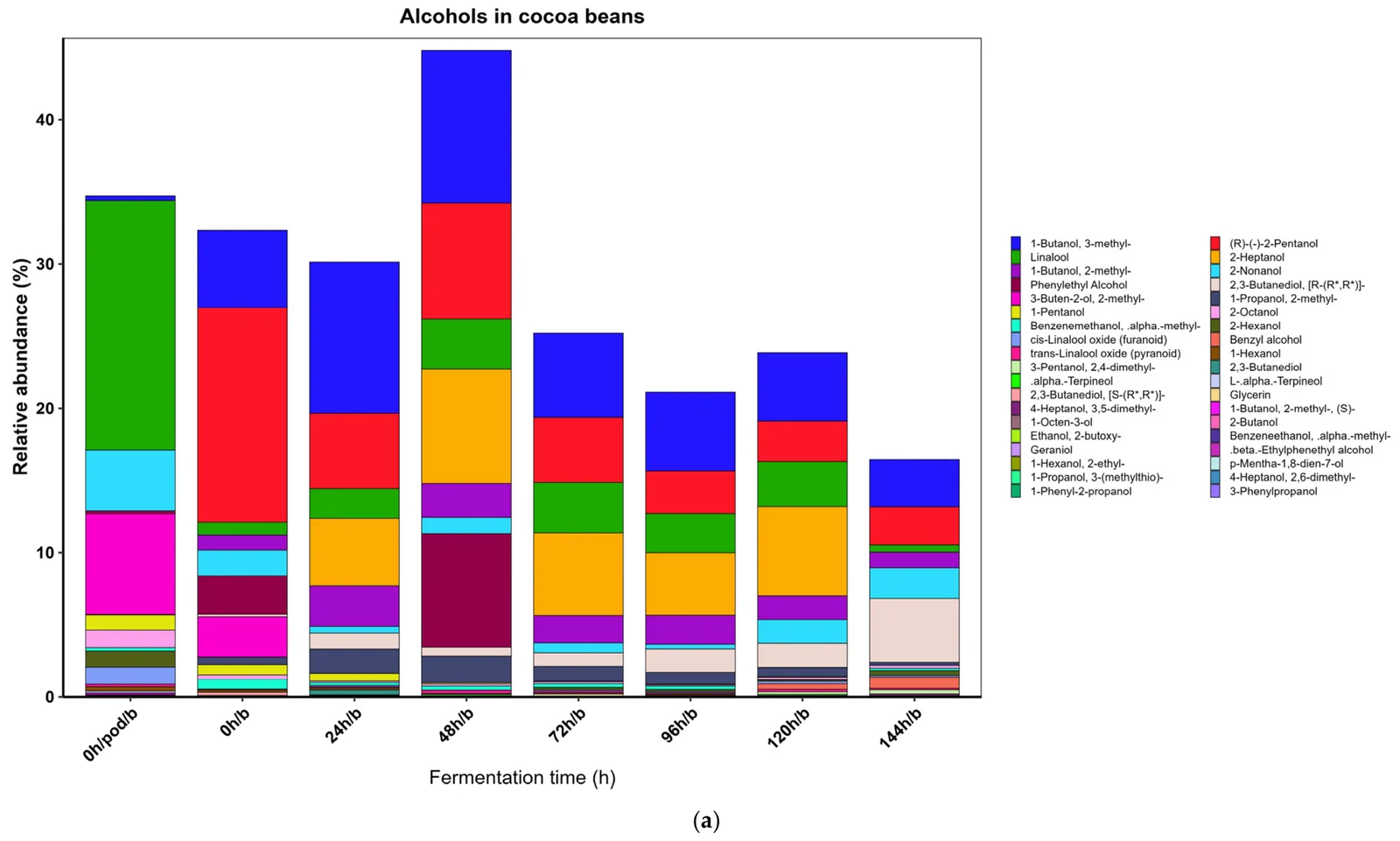

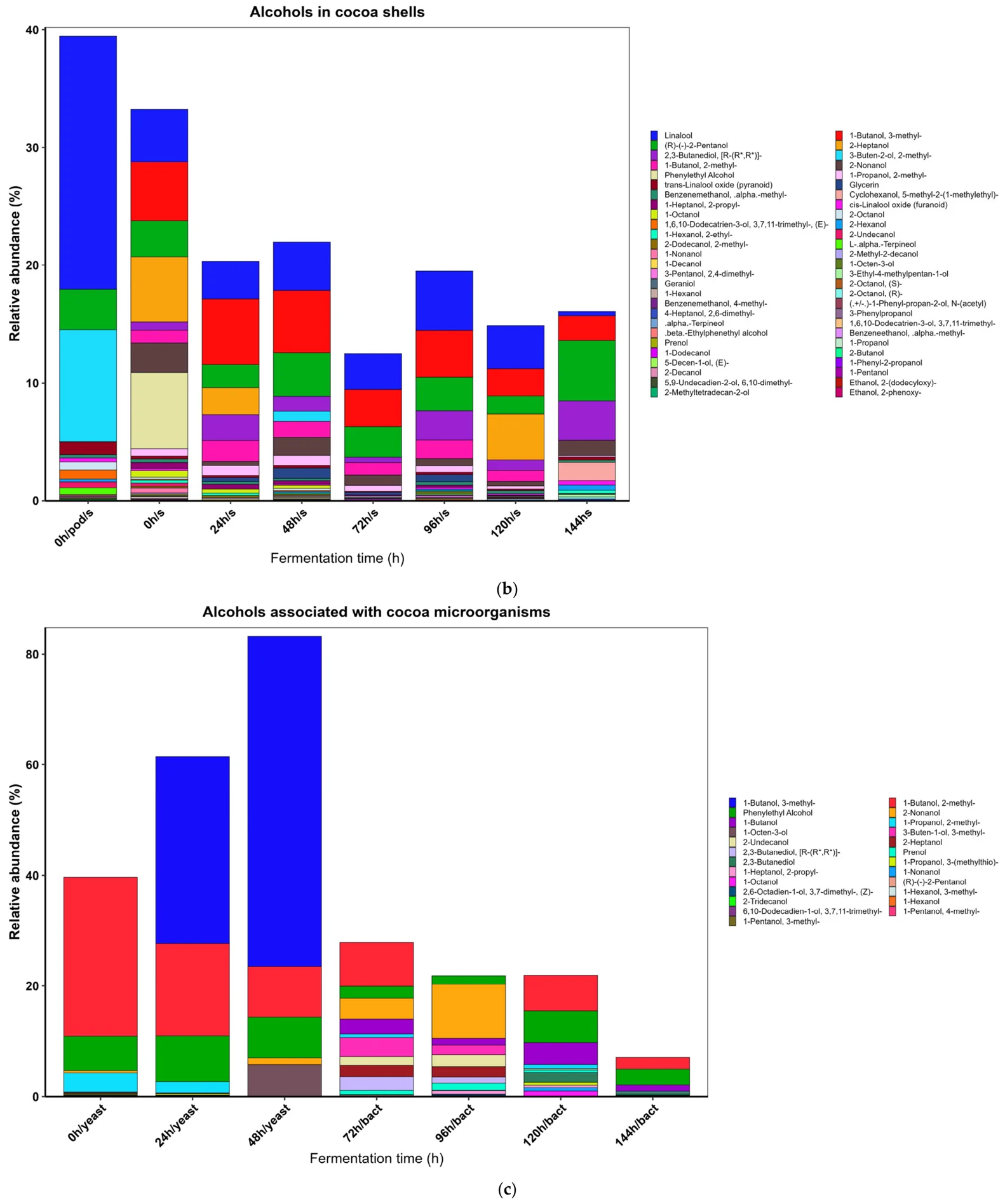
Stacked bar charts showing the relative abundance of volatile compounds within the alcohol family during spontaneous fermentation: (**a**) in the cocoa bean kernel, (**b**) in the cocoa bean shell, and (**c**) associated with isolated cocoa microorganisms.

**Figure 11:**
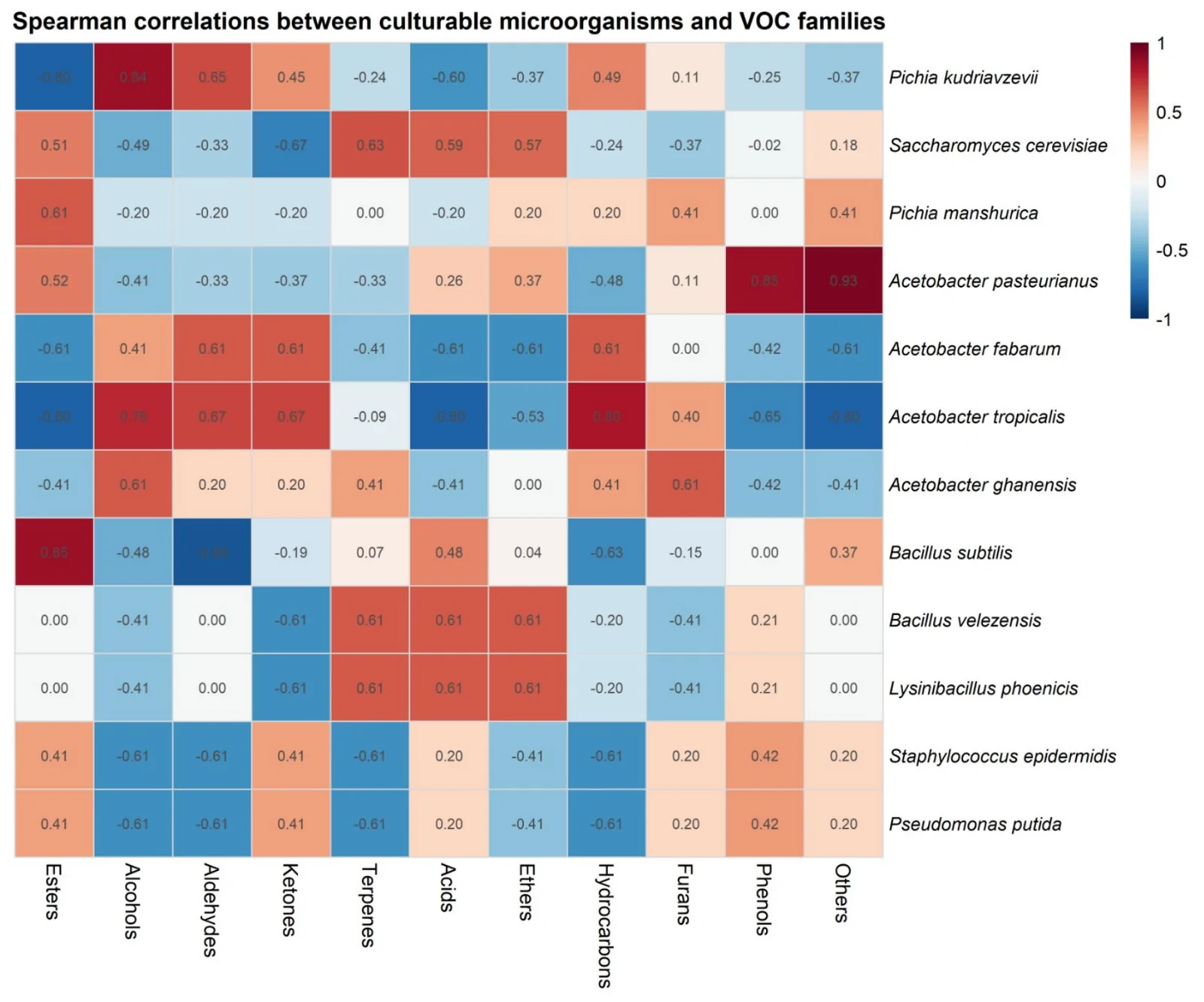
Spearman rank correlations between the recovery frequency of culturable microorganisms and the relative abundance of volatile compound families across the seven fermentation stages. Cell values represent Spearman’s correlation coefficient (ρ). Positive and negative coefficients indicate direct and inverse temporal correlations, respectively. Correlations were calculated across the seven fermentation stages, and *p*-values were adjusted using the Benjamini–Hochberg procedure. None of the associations remained significant after multiple-comparison correction at an FDR threshold of 0.05 (all adjusted *p*-values > 0.05). Accordingly, the correlations shown are descriptive and exploratory and should not be interpreted as evidence of causal relationships between individual microorganisms and volatile compound families.

**Table 1:** Molecular identification of yeasts during the spontaneous fermentation of Criollo cacao.

Sample	Species	GenBank Accession Number	Identity (%)
T_0_A_1_YPD	*Pichia kudriavzevii*	KY104576.1	99.18
T_0_B_2_YPD	*Pichia kudriavzevii*	KY104577.1	100.00
T_0_C_1_YPD	*Pichia kudriavzevii*	KY104577.1	99.84
T_0_D_2_YPD	*Pichia kudriavzevii*	KY104577.1	100.00
T_1_A_2_YPD	*Pichia kudriavzevii*	KY104577.1	100.00
T_1_B_2_YPD	*Pichia kudriavzevii*	KY104577.1	100.00
T_1_C_2_YPD	*Pichia kudriavzevii*	KY104577.1	100.00
T_1_D_2_YPD	*Pichia kudriavzevii*	KY104577.1	100.00
T_2_A_1_YPD	*Pichia kudriavzevii*	KY104577.1	100.00
T_2_B_3_YPD	*Pichia kudriavzevii*	KY104577.1	100.00
T_2_C_2_YPD	*Pichia kudriavzevii*	KY104577.1	100.00
T_2_D_3_YPD	*Pichia kudriavzevii*	KY104577.1	100.00
T_3_A_2_YPD	*Pichia kudriavzevii*	KY104577.1	100.00
T_3_B_2_YPD	*Pichia kudriavzevii*	KY104577.1	100.00
T_3_C_3_YPD	*Pichia kudriavzevii*	KY104577.1	100.00
T_3_D_3_YPD	*Saccharomyces cerevisiae*	KC881067.1	100.00
T_4_A_3_YPD	*Saccharomyces cerevisiae*	KY105046.1	100.00
T_4_B_1_YPD	*Saccharomyces cerevisiae*	MH595372.1	100.00
T_4_C_2_YPD	*Saccharomyces cerevisiae*	OQ085081.1	100.00
T_4_D_2_YPD	*Saccharomyces cerevisiae*	MH595372.1	100.00
T_5_B_3_YPD	*Pichia manshurica*	OM523901.1	99.84
T_5_C_3_YPD	*Pichia manshurica*	OM523901.1	99.84
T_5_D_3_YPD	*Saccharomyces cerevisiae*	MH595372.1	100.00
T_6_A_3_YPD	*Pichia kudriavzevii*	MW856016.1	99.68
T_4_C_3_MRS	*Saccharomyces cerevisiae*	KT726922.1	100.00

**Table 2:** Molecular identification of bacteria during the spontaneous fermentation of Criollo cacao.

Sample	Species	GenBank Accession Number	Identity (%)
T5A2YPD	*Acetobacter pasteurianus*	FN429067.1	100.00
T6B2YPD	*Staphylococcus epidermidis*	FJ613577.1	98.73
T6C2YPD	*Pseudomonas putida*	OP900036.1	100.00
T6D2YPD	*Bacillus subtilis*	KT982213.1	100.00
T0A2MRS	*Acetobacter fabarum*	PX788810.1	99.78
T0B3MRS	*Acetobacter fabarum*	MN909084.1	99.79
T0C1MRS	*Acetobacter tropicalis*	KX424636.1	98.42
T0D3MRS	*Acetobacter tropicalis*	KX424636.1	98.06
T1A2MRS	*Acetobacter pasteurianus*	MZ314699.1	100.00
T1B3MRS	*Acetobacter pasteurianus*	MZ314699.1	100.00
T1C3MRS	*Acetobacter pasteurianus*	MZ314699.1	100.00
T1D3MRS	*Bacillus subtilis*	HQ443231.1	99.80
T2A3MRS	*Acetobacter ghanensis*	NR_113555.1	99.59
T2B2MRS	*Bacillus subtilis*	PQ600452.1	100.00
T2C1MRS	*Acetobacter pasteurianus*	MN909069.1	99.79
T2D1MRS	*Acetobacter tropicalis*	NR_036881.1	99.90
T3A1MRS	*Bacillus subtilis*	PQ600452.1	100.00
T3B3MRS	*Bacillus subtilis*	PQ600452.1	100.00
T3C1MRS	*Bacillus subtilis*	PQ600452.1	100.00
T3D3MRS	*Acetobacter pasteurianus*	KR149362.1	99.90
T4A1MRS	*Bacillus subtilis*	PQ600452.1	100.00
T4B3MRS	*Acetobacter pasteurianus*	NR_117257.1	99.58
T4C3MRS	*Lysinibacillus phoenicis*	EU977816.1	99.48
T4D2MRS	*Bacillus velezensis*	OR144339.1	100.00
T5A3MRS	*Bacillus subtilis*	CP015375.1	100.00
T5B1MRS	*Acetobacter pasteurianus*	NR_117257.1	99.38
T5C1MRS	*Acetobacter pasteurianus*	NR_117257.1	99.38
T5D3MRS	*Acetobacter pasteurianus*	NR_117257.1	99.48
T6A1MRS	*Acetobacter pasteurianus*	NR_117257.1	100.00
T6B1MRS	*Acetobacter pasteurianus*	NR_117257.1	100.00
T6C1MRS	*Bacillus subtilis*	CP015375.1	100.00

## Data Availability

The original contributions presented in this study are included in the article/Supplementary Material. Further inquiries can be directed to the corresponding author.

## Supplementary Materials

The following supporting information can be downloaded at https://www.mdpi.com/article/10.3390/foods15193462/s1, Figure S1: Morphology of fungal and yeast isolates at 0, 24, 48, 72, 96, 120, and 144 h of the Criollo cacao fermentation process, shown without degradation halos (A) and with degradation halos (B), respectively; Figure S2: Morphology of bacterial isolates at 0, 24, 48, 72, 96, 120, and 144 h of the Criollo cacao fermentation process, shown without degradation halos (A) and with degradation halos (B), respectively; Figure S3: Line plots of fungi and yeasts cultured on YPD medium (A) and bacteria cultured on MRS medium (B) during the spontaneous fermentation of Criollo cacao from Lagunas, Cajaruro District, Utcubamba Province, Amazonas Region, Peru; Figure S4: Line plots of fungi and yeasts cultured on YPD medium (A) and bacteria cultured on MRS medium (B) during the spontaneous fermentation of Criollo cacao from Lagunas, Cajaruro District, Utcubamba Province, Amazonas Region, Peru; Table S1: Data on volatile compounds in the nibs of Criollo cocoa beans at 0 h of spontaneous fermentation; Table S2: Exploratory Spearman correlations between culturable microorganisms and volatile compound families during spontaneous Criollo cacao fermentation; Figure S5: Stacked bar chart of the volatile compounds in the acids family present in the cocoa bean’s kernel; Figure S6: Stacked bar chart of the volatile compounds in the acids family present in the cocoa bean’s shell; Figure S7: Stacked bar chart of volatile compounds from the acids family present in cocoa microorganisms; Figure S8: Stacked bar chart of the volatile compounds in the aldehydes family present in the cocoa bean’s kernel; Figure S9. Stacked bar chart of the volatile compounds in the aldehydes family present in the cocoa bean’s shell; Figure S10: Stacked bar chart of volatile compounds from the aldehydes family present in cocoa microorganisms; Figure S11: Stacked bar chart of the volatile compounds in the ketones family present in the cocoa bean’s kernel; Figure S12: Stacked bar chart of the volatile compounds in the ketones family present in the cocoa bean’s shell; Figure S13: Stacked bar chart of volatile compounds from the ketones family present in cocoa microorganisms; Figure S14: Stacked bar chart of the volatile compounds in the terpenes family present in the cocoa bean’s kernel; Figure S15: Stacked bar chart of the volatile compounds in the terpenes family present in the cocoa bean’s shell; Figure S16. Stacked bar chart of volatile compounds from the terpenes family present in cocoa microorganisms.
