# Supplementary material for: Molecular Characterization of Microorganisms, Pectinolytic Potential and Volatile Compound Formation During Spontaneous Fermentation of Criollo Cacao (Theobroma cacao L.)
Source: Foods. 2026 Sep 28;15(19):3462. doi: 10.3390/foods15193462 (PMC13664486; doi:10.3390/foods15193462)
Supplement: Supplementary file 1 [file foods-15-03462-s001.zip › foods-4511683-supplementary.pdf]

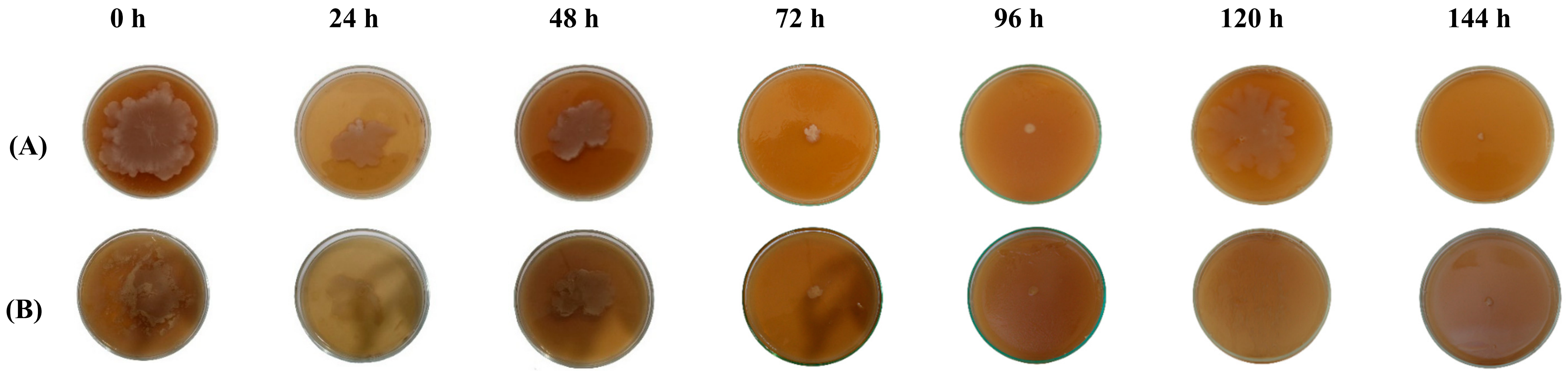

Figure S1. Morphology of fungal and yeast isolates at 0, 24, 48, 72, 96, 120, and 144 h of the Criollo cacao fermentation process, shown without degradation halos (A) and with degradation halos (B), respectively.

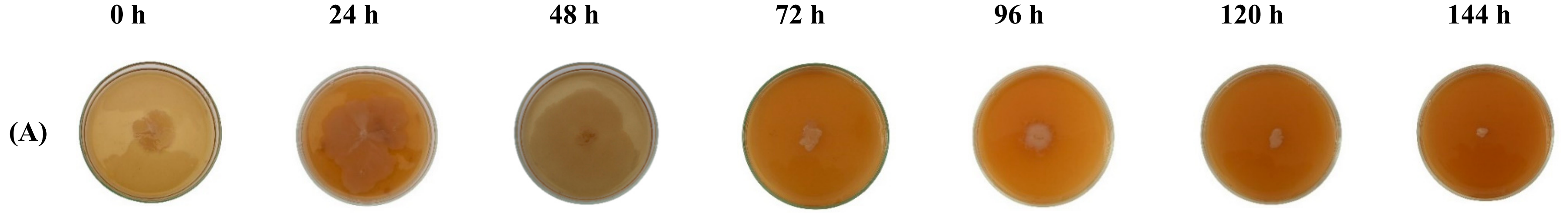

(B)

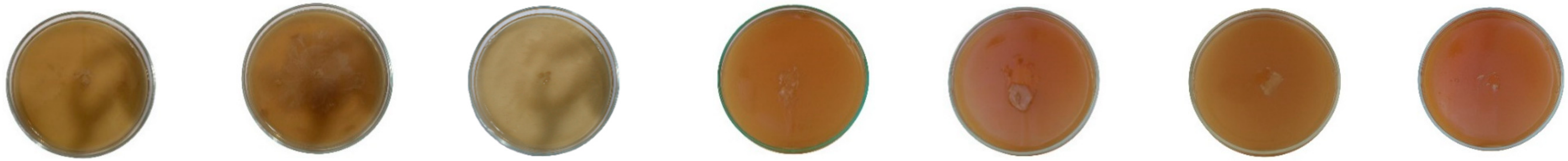

Figure S2. Morphology of bacterial isolates at 0, 24, 48, 72, 96, 120, and 144 h of the Criollo cacao fermentation process, shown without degradation halos (A) and with degradation halos (B), respectively.

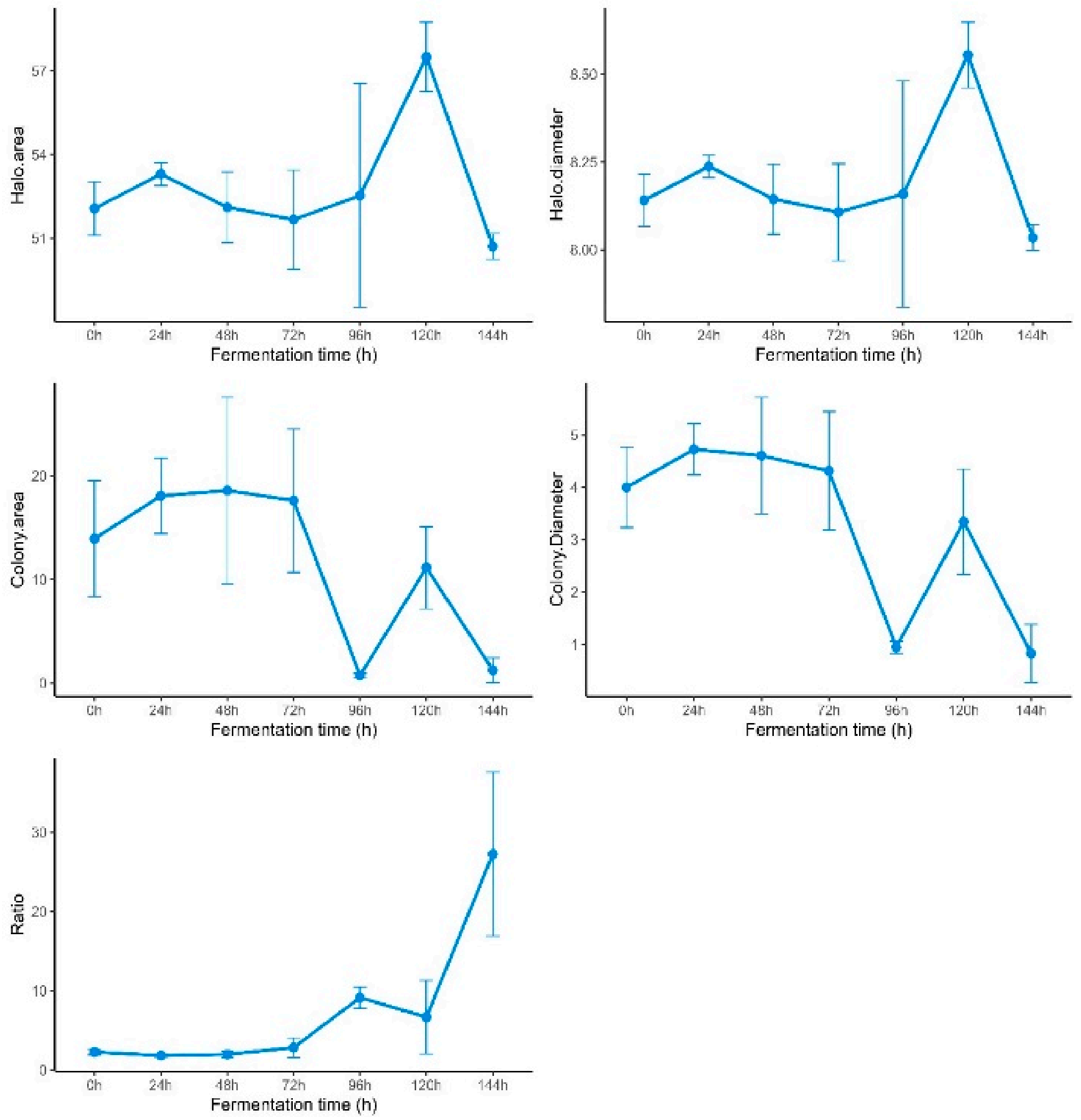

Figure S3. Line plots of fungi and yeasts cultured on YPD medium (A) and bacteria cultured on MRS medium (B) during the spontaneous fermentation of Criollo cacao from Lagunas, Cajaruro District, Utcubamba Province, Amazonas Region, Peru.

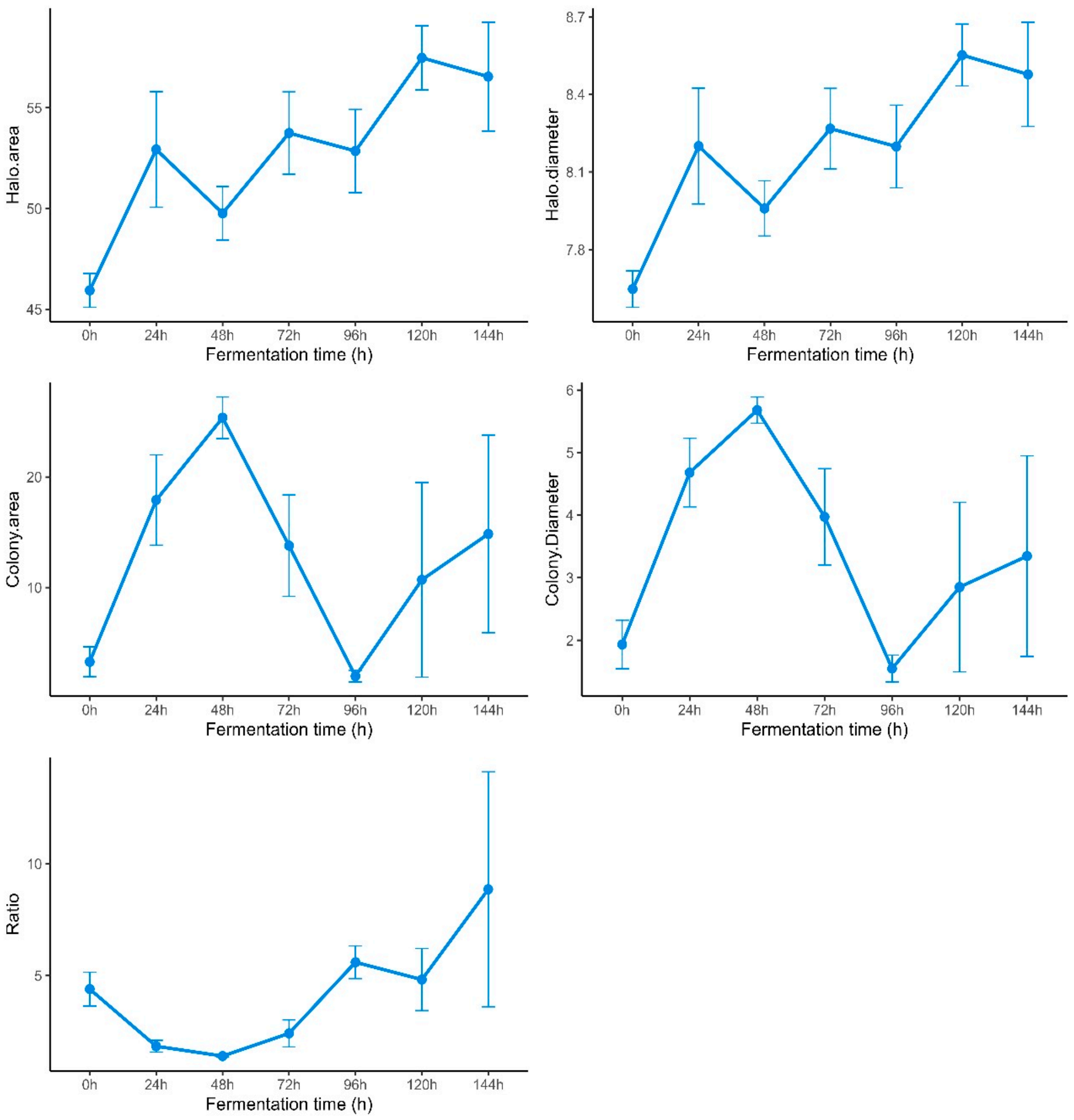

Figure S4. Line plots of fungi and yeasts cultured on YPD medium (A) and bacteria cultured on MRS medium (B) during the spontaneous fermentation of Criollo cacao from Lagunas, Cajaruro District, Utcubamba Province, Amazonas Region, Peru.

Table S1: Data on volatile compounds in the nibs of Criollo cocoa beans at 0 hours of spontaneous fermentation.

File Name	Component RT	Compound Name	Average relative abundance	Standard desviation	Delta RI	Match Factor	Formula	Component RI	Library RI
0h/pod	7.421229591	Propanal, 2-methyl-	1.780387623	0.28399102	-34.7728467	98.9680416	C_4H_8O	586.772847	552
0h/pod	7.694929817	Methacrolein	0.219848999	0.01532485	-25.2600204	85.7358335	C_4H_6O	592.26002	567
0h/pod	8.507574402	2-Butanone	0.046138376	0.00367017	-10.5520129	87.5163009	C_4H_8O	608.552013	598
0h/pod	8.729066456	2-Butanol	0.067842438	0.01809349	-14.9925112	81.3059207	$C_4H_{10}O$	612.992511	598
0h/pod	9.150199844	3-Buten-2-ol, 2-methyl-	6.928011634	0.11697677	-7.43544195	98.0933557	$C_5H_{10}O$	621.435442	614
0h/pod	9.73284986	1-Propanol, 2-methyl-	0.028618988	0.02107123	-8.11647675	86.9292023	$C_4H_{10}O$	633.116477	625
0h/pod	10.75522118	2-Butenal	0.025873534	0.0152738	-24.613095	93.7765809	C_4H_6O	653.613095	629
0h/pod	11.03500389	Butanal, 3-methyl-	4.688660589	0.50098683	-7.22221106	98.7204814	$C_5H_{10}O$	659.222211	652
0h/pod	11.4576997	Butanal, 2-methyl-	3.620110673	0.30408038	-5.69646555	98.2061142	$C_5H_{10}O$	667.696466	662
0h/pod	12.29709924	2-Pentanone	3.560732212	0.43355666	0.47515567	98.6222129	$C_5H_{10}O$	684.524844	685
0h/pod	13.34894719	Acetoin	0.053578079	0.02263509	7.38758649	89.0954179	$C_4H_8O_2$	705.612414	713
0h/pod	14.62100258	1-Butanol, 3-methyl-	0.318340257	0.02416166	4.933619	98.7253477	$C_5H_{12}O$	731.066381	736
0h/pod	14.84496082	1-Butanol, 2-methyl-, (S)-	0.10862789	0.02006129	-38.6046675	89.5578863	$C_5H_{12}O$	735.604668	697
0h/pod	15.16146957	2-Butenal, 2-methyl-	0.617107278	0.07153332	4.0499284	97.9547716	C_5H_8O	741.950072	746
0h/pod	15.71570984	2-Pentenal, (E)-	0.039318816	0.00229172	0.9384555	92.2933421	C_5H_8O	753.061545	754
0h/pod	16.18897118	1-Pentanol	1.076246562	0.12917618	2.45045751	99.5849227	$C_5H_{12}O$	762.549543	765
0h/pod	16.68366618	Toluene	0.057715212	0.00922367	-9.46724494	96.4169572	C_7H_8	772.467245	763

0h/pod	17.17907187	Furan, 2,3-dihydro-4-methyl-	0.021853657	0.00055373	-21.3940003	87.5946651	C_5H_8O	782.394	761
0h/pod	17.37194484	2-Hexanone	0.623112899	0.04871115	3.73406495	99.1576186	$C_6H_{12}O$	786.265935	790
0h/pod	17.9294838	2-Hexanol	1.121815711	0.0614312	3.55645946	83.0725669	$C_6H_{14}O$	797.443541	801
0h/pod	17.97419974	Furan, 2-ethyl-5-methyl-	0.11901036	0.02053573	3.65998922	84.12292	$C_7H_{10}O$	798.340011	802
0h/pod	18.0162795	Hexanal	1.753720055	0.08921072	0.81636935	95.1921305	$C_6H_{12}O$	799.183631	800
0h/pod	18.85346337	2-Pentanone, 4-hydroxy-	0.009222847	0.00296862	6.03241031	76.7601005	$C_5H_{10}O_2$	815.96759	822
0h/pod	20.22663225	2-Pentanol, acetate	0.006032822	0.00165084	5.50296215	78.7825009	$C_7H_{14}O_2$	843.497038	849
0h/pod	20.75619144	2-Hexenal, (E)-	0.027224143	0.00728491	-0.11370166	87.0119499	$C_6H_{10}O$	854.113702	854
0h/pod	21.31274313	1-Hexanol	0.279128899	0.04608819	2.72848571	98.7728219	$C_6H_{14}O$	865.271514	868
0h/pod	21.61012552	4-Heptanone	0.058773298	0.00346403	0.76652928	95.741182	$C_7H_{14}O$	871.233471	872
0h/pod	21.72587058	5-Hepten-2-one	0.265655945	0.01115103	-7.55394114	87.5665058	$C_7H_{12}O$	873.553941	866
0h/pod	22.60113181	4-Hepten-2-one, (E)-	0.280599906	0.01906188	-30.1012792	80.9949011	$C_7H_{12}O$	891.101279	861
0h/pod	23.4490884	Methional	0.102085458	0.01050877	-1.5939686	91.8561865	C_4H_8OS	908.593969	907
0h/pod	24.68527105	3-Hepten-2-one	0.055030853	0.00230702	2.11546045	95.9050926	$C_7H_{12}O$	934.88454	937
0h/pod	25.23790215	Bicyclo[3.1.0]hex-2-ene, 2-methyl-5-(1-methylethyl)-	0.005271523	0.00022927	-17.6376468	75.8199624	$C_{10}H_{16}$	946.637647	929
0h/pod	25.70746376	Hexanoic acid	0.106847621	0.04047145	33.3759303	95.9103875	$C_6H_{12}O_2$	956.62407	990
0h/pod	25.80824439	2-Heptenal, (E)-	0.149532216	0.03452444	-0.76742649	97.0124654	$C_7H_{12}O$	958.767427	958
0h/pod	26.04086571	Octane, 2,2,6-trimethyl-	0.009544538	0.00186683	-0.714711	72.9621343	$C_{11}H_{24}$	963.714711	963
0h/pod	26.20121264	Cyclotetrasiloxane, octamethyl-	2.186984249	1.02498102	26.8751034	95.4894442	$C_8H_{24}O_4Si_4$	967.124897	994

0h/pod	26.34242445	Heptan-2-yl formate	0.009316905	0.00096335	-1.1281252	83.260654	$C_8H_{16}O_2$	970.128125	969
0h/pod	26.47203085	Benzaldehyde	1.644807878	0.29570961	-10.8845353	99.2862721	C_7H_6O	972.884535	962
0h/pod	26.73646535	1-Octen-3-ol	0.040749431	0.01382482	1.49159194	84.4278457	$C_8H_{16}O$	978.508408	980
0h/pod	27.12675487	2,6-Dimethyl-2-trans-6-octadiene	0.003849369	0.00047729	4.19109168	76.9464327	$C_{10}H_{18}$	986.808908	991
0h/pod	27.22899487	2-Octanone	1.62629366	0.064746	1.0166978	97.3600318	$C_8H_{16}O$	988.983302	990
0h/pod	27.32116158	Cyclopropane, 1,1-dimethyl-2-(2,4-pentadienyl)-	9.260173559	0.53207908	-39.9434619	83.6500367	$C_{10}H_{16}$	990.943462	951
0h/pod	27.41348904	Furan, 2-pentyl-	0.224866456	0.05615477	0.09295951	91.1301058	$C_9H_{14}O$	992.907041	993
0h/pod	27.46207261	Hexanoic acid, ethyl ester	0.107134016	0.05822476	6.0597062	88.996515	$C_8H_{16}O_2$	993.940294	1000
0h/pod	27.55892971	(2R,5R)-2-Methyl-5-(prop-1-en-2-yl)-2-vinyltetrahydrofuran	0.05667272	0.00590477	-2.00020658	83.8887236	$C_{10}H_{16}O$	996.000207	994
0h/pod	27.67140248	2-Octanol	1.215579524	0.03839826	-0.39222626	98.5658665	$C_8H_{18}O$	998.392226	998
0h/pod	27.8754357	3-Carene	0.02912295	0.00240413	8.05827531	83.6530883	$C_{10}H_{16}$	1002.94173	1011
0h/pod	27.89907021	Octanal	0.099240005	0.00976419	-0.48305571	97.2189302	$C_8H_{16}O$	1003.48306	1003
0h/pod	28.24349307	(2R,5S)-2-Methyl-5-(prop-1-en-2-yl)-2-vinyltetrahydrofuran	0.158504956	0.0092107	-3.37180656	94.5576716	$C_{10}H_{16}O$	1011.37181	1008
0h/pod	28.53677992	.alpha.-Phellandrene	0.04838618	0.00958334	-13.0893249	91.4777721	$C_{10}H_{16}$	1018.08933	1005
0h/pod	28.69636619	3,6-Heptanedione	0.325373447	0.05510023	19.2554698	93.4975703	$C_7H_{12}O_2$	1021.74453	1041
0h/pod	29.20053811	2-Heptanol, acetate	0.336633778	0.07381167	11.7077848	98.328711	$C_9H_{18}O_2$	1033.29222	1045

0h/pod	29.33050569	trans-.beta.-Ocimene	11.89874252	0.4169658	12.7309738	98.5573987	$C_{10}H_{16}$	1036.26903	1049
0h/pod	29.5691076	D-Limonene	0.331314589	0.02086253	-23.7340266	94.9278274	$C_{10}H_{16}$	1041.73403	1018
0h/pod	29.86770609	1,3,6-Octatriene, 3,7-dimethyl-, (Z)-	2.115647465	0.22403012	-10.5732041	98.3811579	$C_{10}H_{16}$	1048.5732	1038
0h/pod	30.12650532	Benzeneacetaldehyde	6.666083215	0.38252893	-9.50080894	97.9966933	C_8H_8O	1054.50081	1045
0h/pod	30.35105001	.gamma.-Terpinene	0.037601191	0.00712557	0.35616106	88.9696704	$C_{10}H_{16}$	1059.64384	1060
0h/pod	30.41914486	2-Octenal, (E)-	0.13239934	0.02573601	-1.2035012	94.3481161	$C_8H_{14}O$	1061.2035	1060
0h/pod	30.7415618	Benzenemethanol, .alpha.-methyl-	0.230368416	0.03772374	-7.58822269	92.8177839	$C_8H_{10}O$	1068.58822	1061
0h/pod	31.02440185	3-Oxatricyclo[4.1.1.0(2,4)]octane, 2,7,7-trimethyl-	0.140761884	0.0106008	19.9335353	86.2432238	$C_{10}H_{16}O$	1075.06647	1095
0h/pod	31.17359344	Acetophenone	1.058738822	0.25276657	-13.4835878	98.3472105	C_8H_8O	1078.48359	1065
0h/pod	31.28764262	2-Furanmethanol, 5-ethenyltetrahydro-.alpha.,.alpha.,5-trimethyl-, cis-	1.18010265	0.07825149	-7.09579974	98.6432665	$C_{10}H_{18}O_2$	1081.0958	1074
0h/pod	31.60589272	2-Methyl-6-methyleneoct-7-en-4-one	0.00921451	0.00040791	-4.38508297	79.0946544	$C_{10}H_{16}O$	1088.38508	1084
0h/pod	31.95012941	trans-Linalool oxide (furanoid)	1.404519326	0.09939273	-10.2695697	98.5920391	$C_{10}H_{18}O_2$	1096.26957	1086
0h/pod	32.08506321	2-Nonanol	4.222404985	0.39106067	2.63987156	99.1953626	$C_9H_{20}O$	1099.36013	1102
0h/pod	32.19198091	Linalool	17.2894828	1.15608166	-2.94630143	99.2700375	$C_{10}H_{18}O$	1101.9463	1099
0h/pod	32.3530445	Nonanal	0.27718585	0.03945171	-1.91534007	97.8710779	$C_9H_{18}O$	1105.91534	1104

0h/pod	33.10458654	Phenylethyl Alcohol	0.256121996	0.04646205	-8.43535087	97.9349728	$C_8H_{10}O$	1124.43535	1116
0h/pod	33.38231852	2,4,6-Octatriene, 2,6-dimethyl-, (E,Z)-	5.105262421	0.20338186	-0.2794116	98.735264	$C_{10}H_{16}$	1131.27941	1131
0h/pod	33.64912623	p-Mentha-1,5,8-triene	0.254471952	0.02891077	-26.8542688	96.6386416	$C_{10}H_{14}$	1137.85427	1111
0h/pod	34.6941892	Isoneral	0.054828879	0.01146784	6.39257764	89.4668951	$C_{10}H_{16}O$	1163.60742	1170
0h/pod	35.53697383	(3R,6S)-2,2,6-Trimethyl-6-vinyltetrahydro-2H-pyran-3-ol	0.182392521	0.02703791	-11.3758952	91.5039458	$C_{10}H_{18}O_2$	1184.3759	1173
0h/pod	35.83986867	2-Decanone	0.019784221	0.00558354	1.15996388	77.6977119	$C_{10}H_{20}O$	1191.84004	1193
0h/pod	36.5917744	L-.alpha.-Terpineol	0.133443389	0.02678726	-21.1110219	96.4961815	$C_{10}H_{18}O$	1211.11102	1190
0h/pod	37.19930861	2-Nonanol, acetate	0.557399345	0.08928404	9.84635304	98.5162202	$C_{11}H_{22}O_2$	1227.15365	1237
0h/pod	38.04977734	Linalyl acetate	0.134200897	0.04070596	7.38876842	94.335567	$C_{12}H_{20}O_2$	1249.61123	1257
0h/pod	38.55958589	Acetic acid, 2-phenylethyl ester	0.154491679	0.03922323	-5.07330052	96.8861111	$C_{10}H_{12}O_2$	1263.0733	1258
0h/pod	38.72134898	2-Decenal, (Z)-	0.052491712	0.00559733	-15.3668503	73.435057	$C_{10}H_{18}O$	1267.36685	1252
0h/pod	38.72180277	2-Decenal, (E)-	0.066095754	0.02136283	-4.35345599	82.9596892	$C_{10}H_{18}O$	1267.35346	1263
0h/pod	38.95316952	2,6-Octadienal, 3,7-dimethyl-, (E)-	0.018001986	0.00474358	-3.46631941	85.6055953	$C_{10}H_{16}O$	1273.46632	1270
0h/pod	38.96105677	Citral	0.018881708	0.00199266	2.37399503	83.6198987	$C_{10}H_{16}O$	1273.62601	1276
0h/pod	39.15976692	(Z)-Undec-6-en-2-one	0.147284797	0.07703132	-4.92175654	86.71113	$C_{11}H_{20}O$	1278.92176	1274
0h/pod	39.70747024	2-Undecanone	0.02862814	0.00767725	0.61552044	86.8547664	$C_{11}H_{22}O$	1293.38448	1294
0h/pod	39.88842916	1-Cyclohexene-1-carboxaldehyde,	0.017033915	0.00040102	-26.1629036	86.6873996	$C_{10}H_{14}O$	1298.1629	1272

		4-(1-methylethenyl)-							
0h/pod	40.54657458	p-Mentha-1,8-dien-7-ol	0.045814515	0.00288692	-20.60295	90.9089291	$C_{10}H_{16}O$	1316.60295	1296
0h/pod	40.76798566	trans-Geranic acid methyl ester	0.03293601	0.00954473	1.1513213	89.360151	$C_{11}H_{18}O_2$	1322.84868	1324
0h/pod	43.69059131	Benzoic acid, pent-2-yl ester	0.071767768	0.01487601	-11.6283022	91.9535686	$C_{12}H_{16}O_2$	1405.6283	1394
0h/pod	46.72982063	Heptasiloxane, 1,1,3,3,5,5,7,7,9,9,11,11,13,13-tetradecamethyl-	0.03920802	0.0058465	29.1856997	76.8779739	$C_{14}H_{44}O_6Si_7$	1496.8143	1526
0h/pod	49.97551811	Benzoic acid, 2,4-dimethylpent-3-yl ester	0.009579823	0.00384781	-22.9528211	72.8580946	$C_{14}H_{20}O_2$	1599.95282	1577

Table S2: Exploratory Spearman correlations between culturable microorganisms and volatile compound families during spontaneous Criollo cacao fermentation.

Culturable microorganism	VOC family	ρ	*p*-value	FDR (BH)
Acetobacter pasteurianus	Others	0.927	0.0027	0.3561
Bacillus subtilis	Esters	0.852	0.0148	0.3973
Bacillus subtilis	Aldehydes	−0.852	0.0148	0.3973
Acetobacter pasteurianus	Phenols	0.846	0.0164	0.3973
Pichia kudriavzevii	Alcohols	0.842	0.0175	0.3973
Pichia kudriavzevii	Esters	−0.805	0.0291	0.3973
Acetobacter tropicalis	Esters	−0.802	0.0301	0.3973
Acetobacter tropicalis	Acids	−0.802	0.0301	0.3973
Acetobacter tropicalis	Others	−0.802	0.0301	0.3973
Acetobacter tropicalis	Hydrocarbons	0.802	0.0301	0.3973
Acetobacter tropicalis	Alcohols	0.757	0.0487	0.4314

Note: $n = 7$ fermentation time points. Correlations were calculated using Spearman's rank correlation coefficient. FDR values were obtained using the Benjamini–Hochberg procedure. None of the associations remained significant after FDR correction ($q < 0.05$). Therefore, nominal associations are interpreted as exploratory temporal relationships rather than evidence of direct microbial causation.

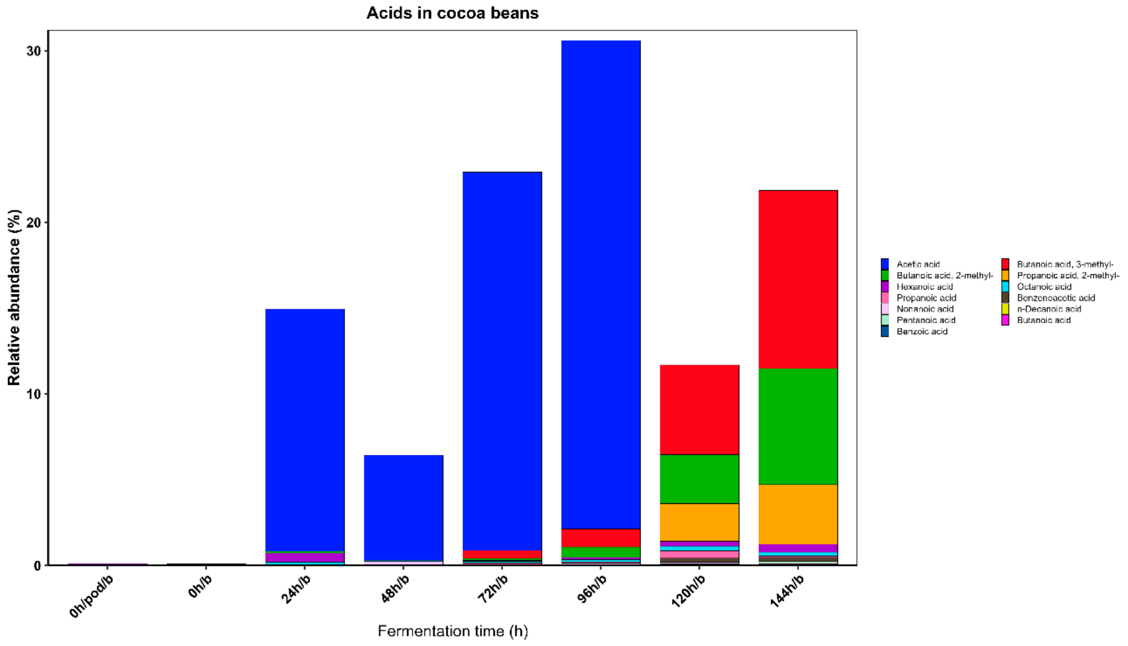

Figure S5. Stacked bar chart of the volatile compounds in the acids family present in the cocoa bean's kernel.

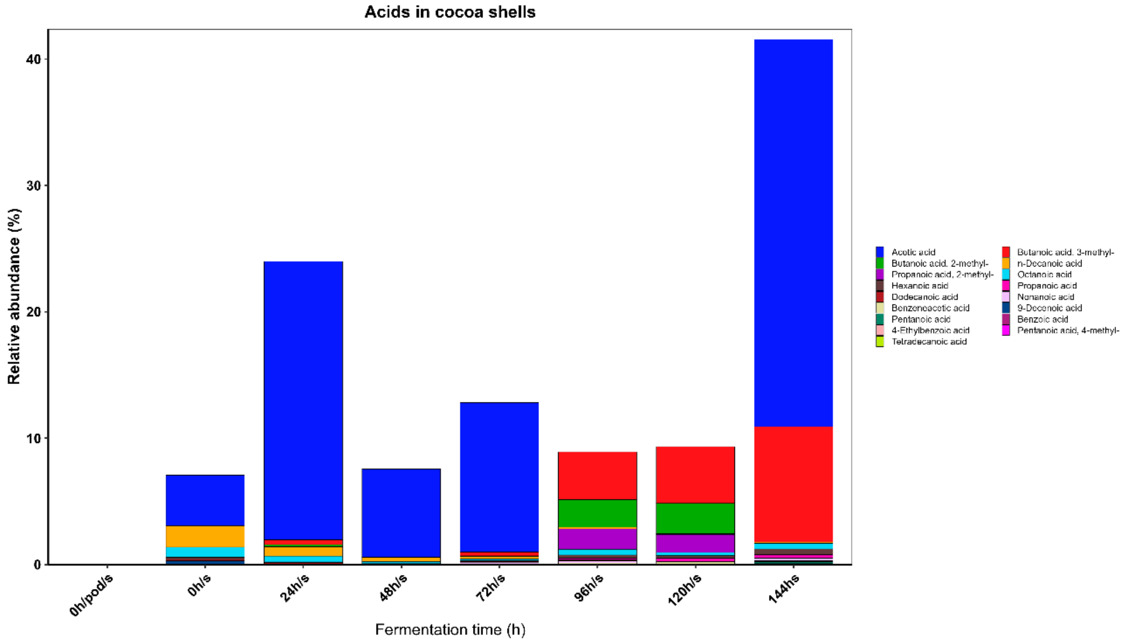

Figure S6. Stacked bar chart of the volatile compounds in the acids family present in the cocoa bean's shell.

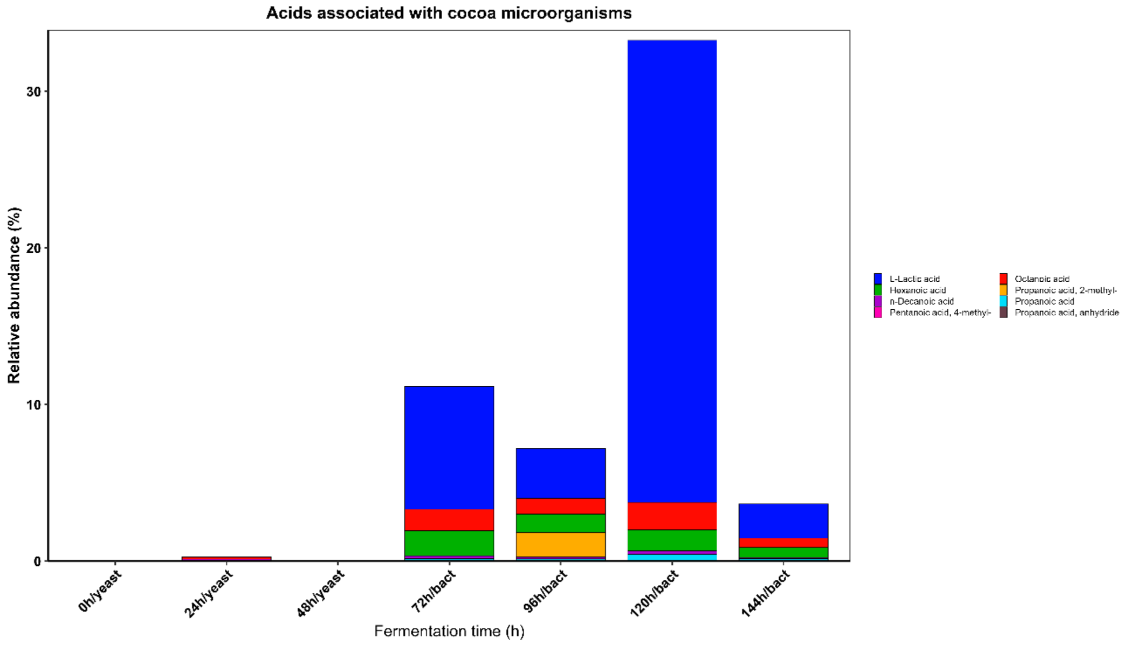

Figure S7. Stacked bar chart of volatile compounds from the acids family present in cocoa microorganisms.

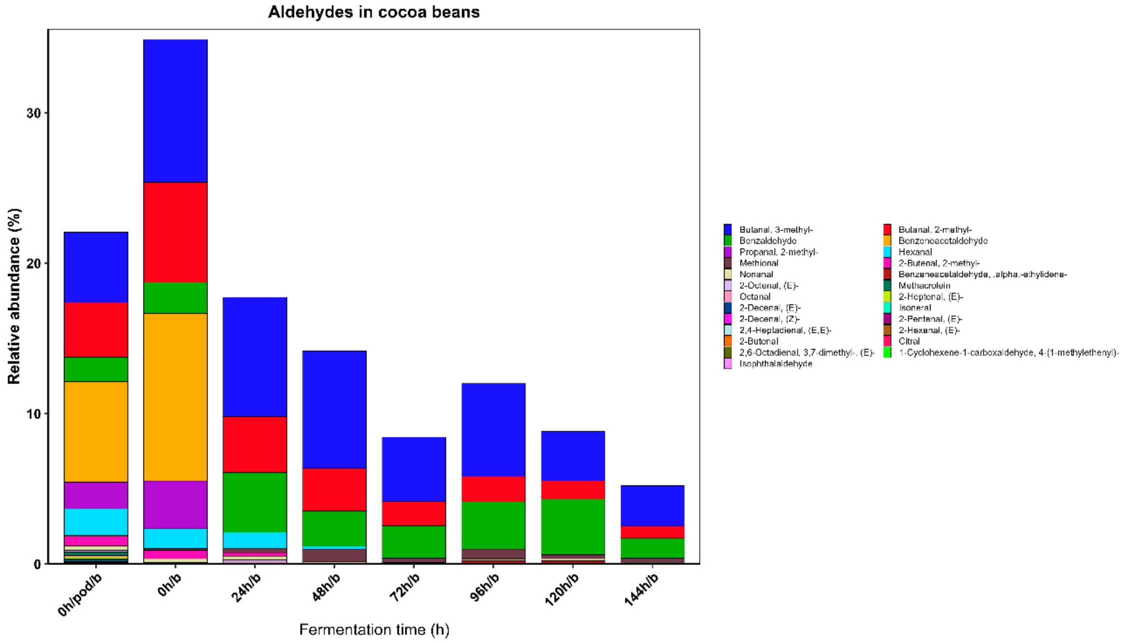

Figure S8. Stacked bar chart of the volatile compounds in the aldehydes family present in the cocoa bean's kernel.

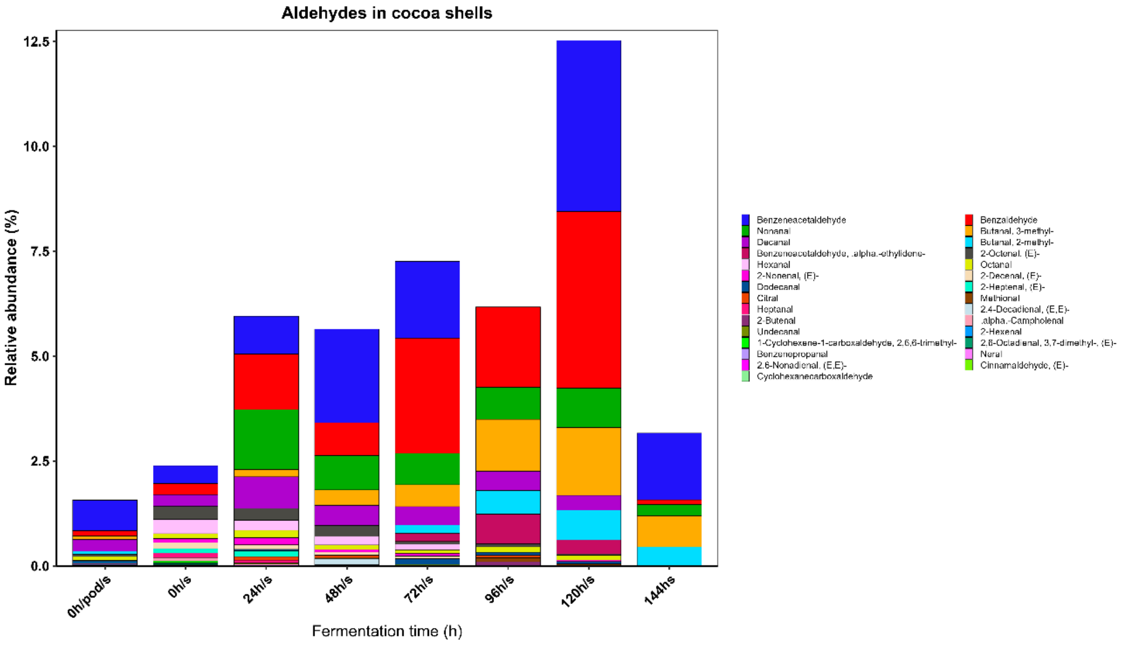

Figure S9. Stacked bar chart of the volatile compounds in the aldehydes family present in the cocoa bean's shell.

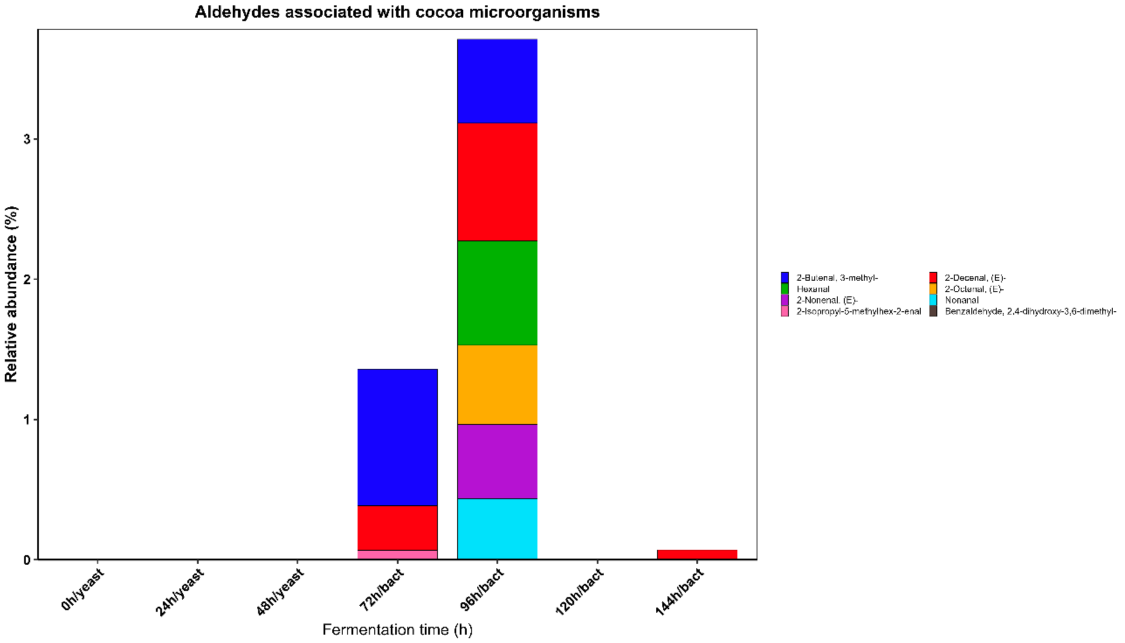

Figure S10. Stacked bar chart of volatile compounds from the aldehydes family present in cocoa microorganisms.

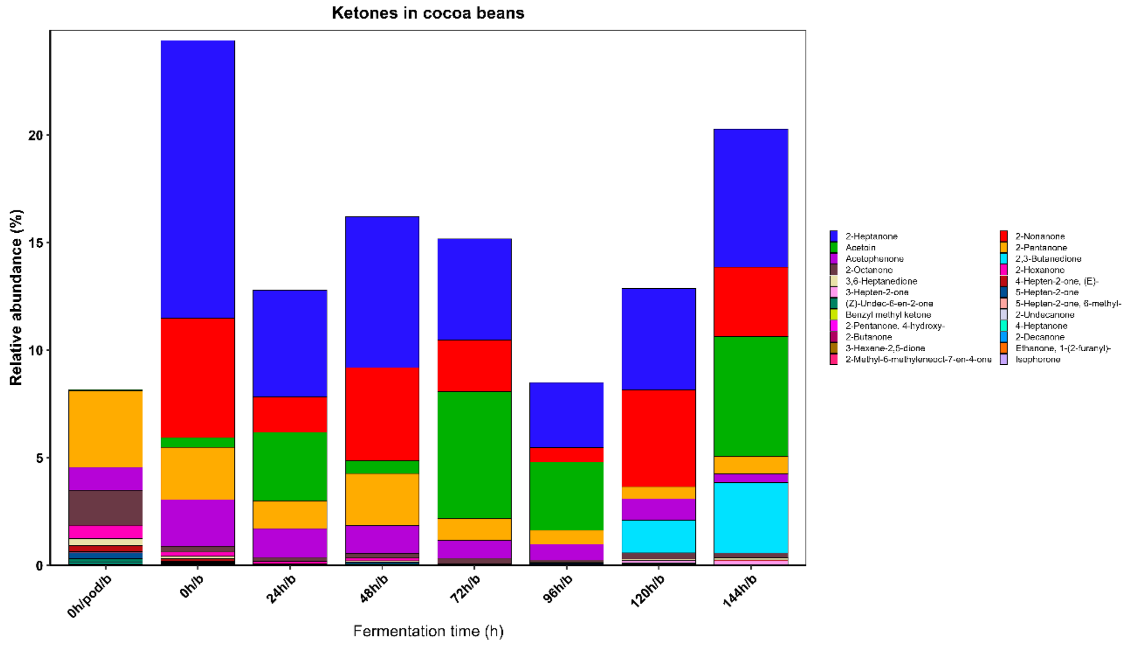

Figure S11. Stacked bar chart of the volatile compounds in the ketones family present in the cocoa bean's kernel.

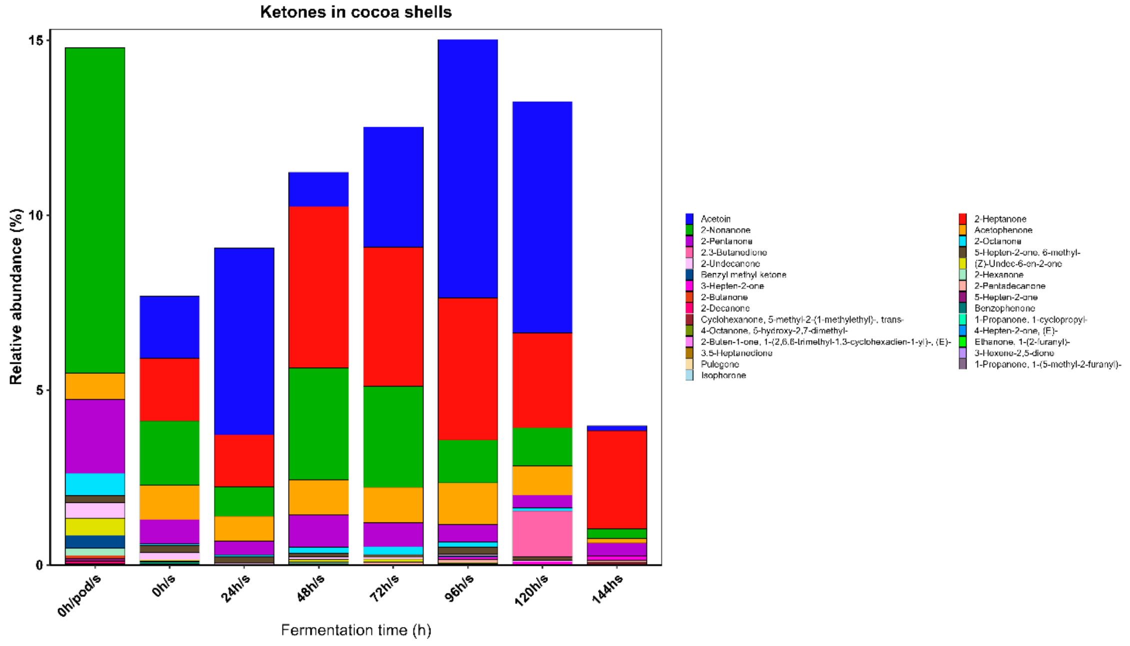

Figure S12. Stacked bar chart of the volatile compounds in the ketones family present in the cocoa bean's shell.

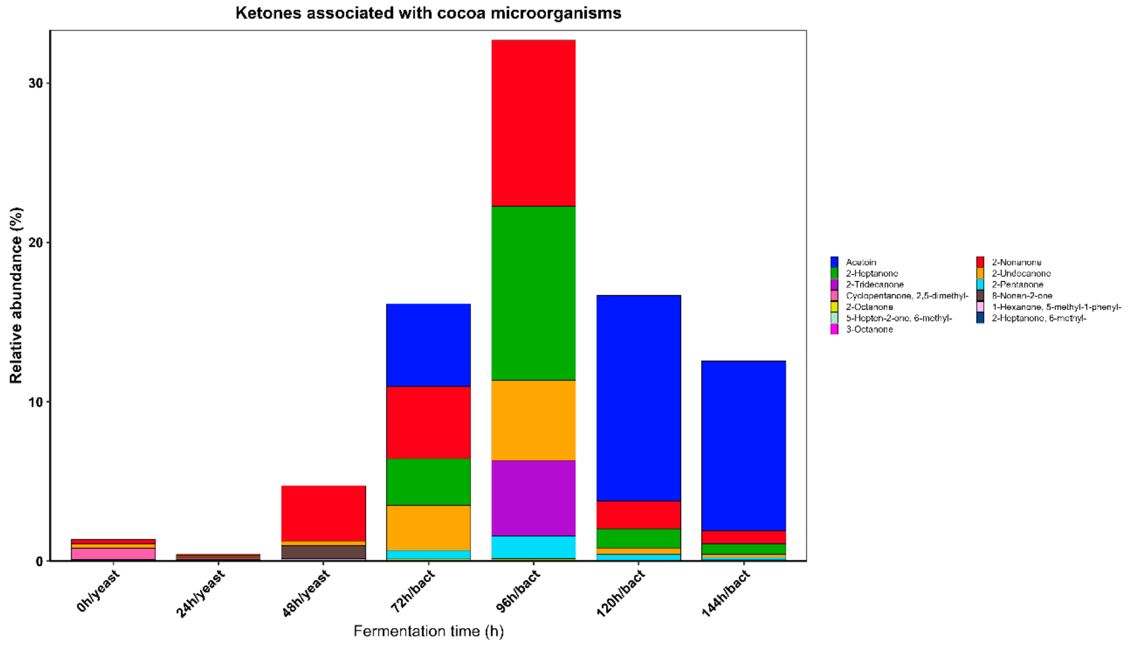

Figure S13. Stacked bar chart of volatile compounds from the ketones family present in cocoa microorganisms.

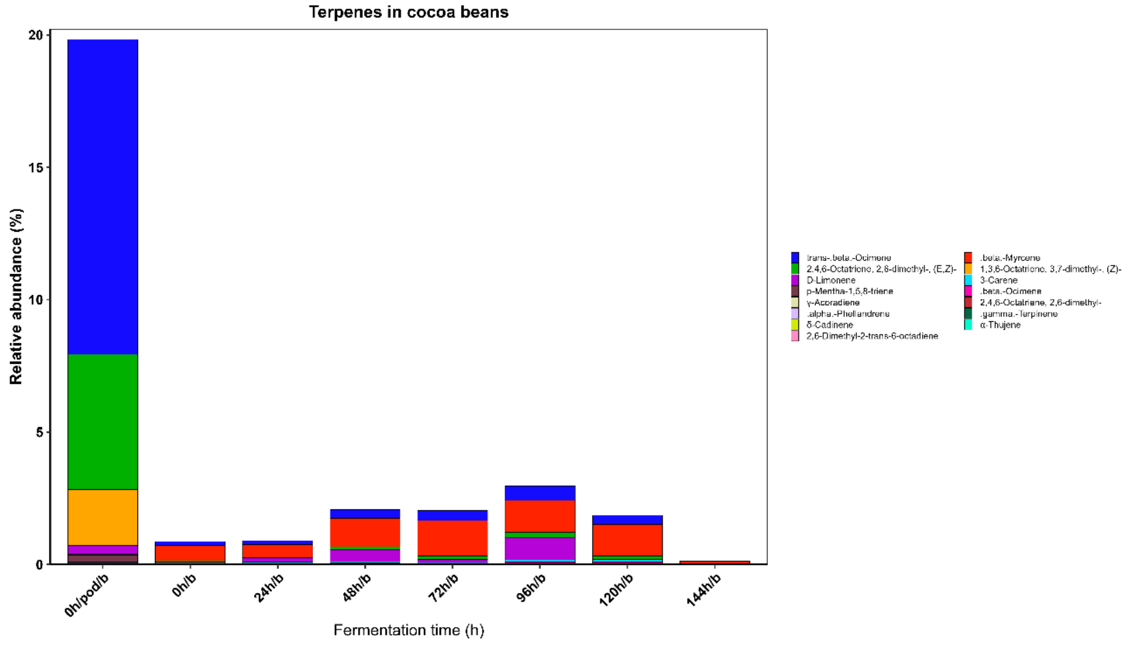

Figure S14. Stacked bar chart of the volatile compounds in the terpenes family present in the cocoa bean's kernel.

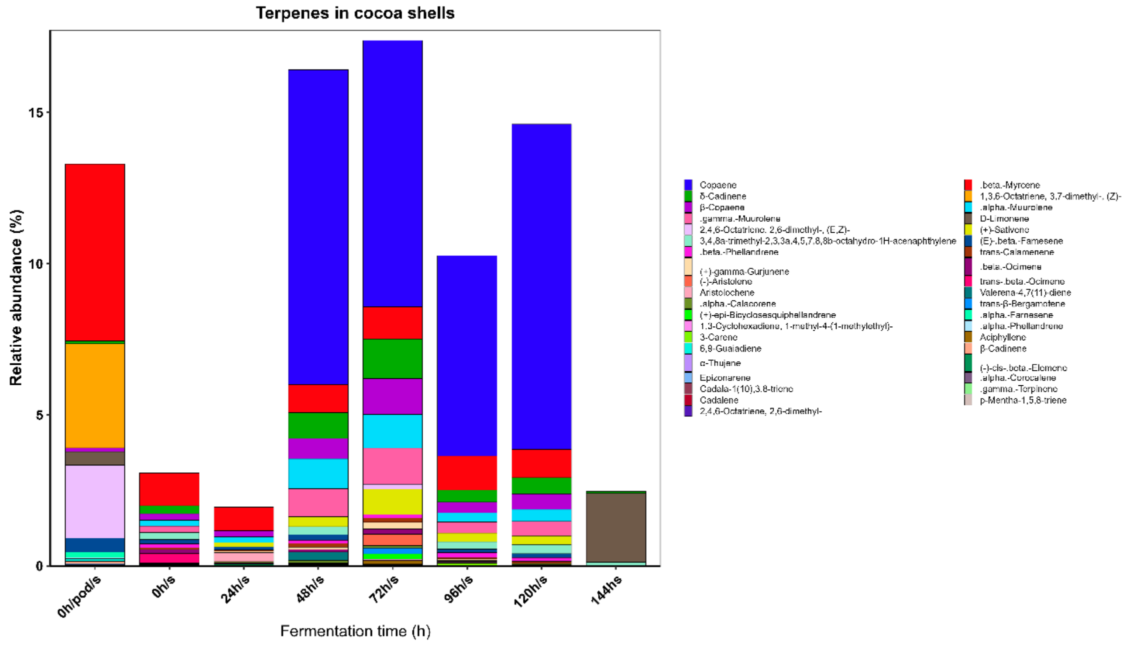

Figure S15. Stacked bar chart of the volatile compounds in the terpenes family present in the cocoa bean's shell.

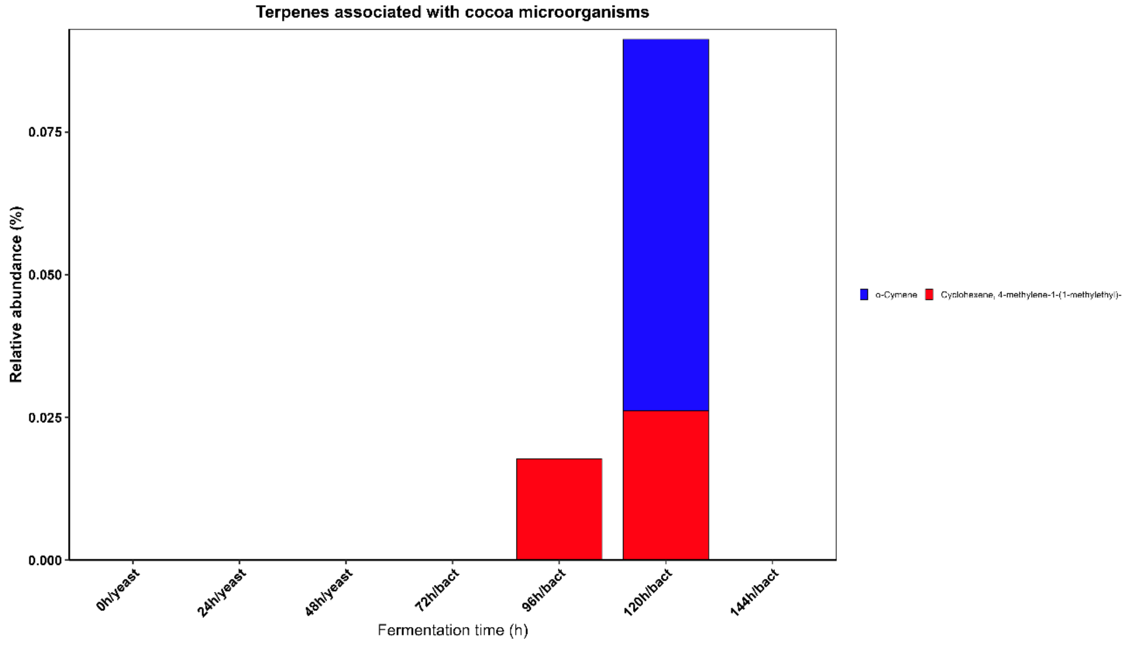

Figure S16. Stacked bar chart of volatile compounds from the terpenes family present in cocoa microorganisms.
